# CD36 and Fyn Kinase Mediate Malaria-Induced Lung Endothelial Barrier Dysfunction in Mice Infected with *Plasmodium berghei*

**DOI:** 10.1371/journal.pone.0071010

**Published:** 2013-08-15

**Authors:** Ifeanyi U. Anidi, Laura E. Servinsky, Otgonchimeg Rentsendorj, R. Scott Stephens, Alan L. Scott, David B. Pearse

**Affiliations:** 1 Department of Molecular Microbiology and Immunology, Bloomberg School of Public Health, Baltimore, Maryland, United States of America; 2 Division of Pulmonary and Critical Care Medicine, Department of Medicine, The Johns Hopkins Medical Institutions, Baltimore, Maryland, United States of America; University of Heidelberg Medical School, Germany

## Abstract

Severe malaria can trigger acute lung injury characterized by pulmonary edema resulting from increased endothelial permeability. However, the mechanism through which lung fluid conductance is altered during malaria remains unclear. To define the role that the scavenger receptor CD36 may play in mediating this response, C57BL/6J (WT) and CD36−/− mice were infected with *P. berghei* ANKA and monitored for changes in pulmonary endothelial barrier function employing an isolated perfused lung system. WT lungs demonstrated a >10-fold increase in two measures of paracellular fluid conductance and a decrease in the albumin reflection coefficient (σ_alb_) compared to control lungs indicating a loss of barrier function. In contrast, malaria-infected CD36−/− mice had near normal fluid conductance but a similar reduction in σ_alb_. In WT mice, lung sequestered iRBCs demonstrated production of reactive oxygen species (ROS). To determine whether knockout of CD36 could protect against ROS-induced endothelial barrier dysfunction, mouse lung microvascular endothelial monolayers (MLMVEC) from WT and CD36−/− mice were exposed to H_2_O_2_. Unlike WT monolayers, which showed dose-dependent decreases in transendothelial electrical resistance (TER) from H_2_O_2_ indicating loss of barrier function, CD36−/− MLMVEC demonstrated dose-dependent increases in TER. The differences between responses in WT and CD36−/− endothelial cells correlated with important differences in the intracellular compartmentalization of the CD36-associated Fyn kinase. Malaria infection increased total lung Fyn levels in CD36−/− lungs compared to WT, but this increase was due to elevated production of the inactive form of Fyn further suggesting a dysregulation of Fyn-mediated signaling. The importance of Fyn in CD36-dependent endothelial signaling was confirmed using *in vitro* Fyn knockdown as well as Fyn−/− mice, which were also protected from H_2_O_2_- and malaria-induced lung endothelial leak, respectively. Our results demonstrate that CD36 and Fyn kinase are critical mediators of the increased lung endothelial fluid conductance caused by malaria infection.

## Introduction

Severe malaria, a major source of morbidity and mortality in the developing world, is frequently complicated by acute respiratory distress syndrome (ARDS) which is characterized by increased vascular permeability resulting in pulmonary edema [1]. ARDS has been reported for all five species of *Plasmodium* that infect humans and is considered an important predictor of disease mortality [2]. Despite this, the mechanisms that drive *Plasmodium*-mediated pulmonary injury remain poorly understood [2].

The etiological agents of human malaria are vector-borne protozoan parasites that initially infect liver cells but rapidly develop to invade and reproduce in host erythrocytes. These blood-stage parasites produce a number of proteins that are exported to the surface of the infected red blood cell (iRBC) [3]. For *P. falciparium*, one of these exported proteins, erythrocyte membrane protein 1 (PfEMP1), interacts with host ligands including the scavenger receptor CD36, the adhesion molecules ICAM1 and PCAM-1 and the extracellular matrix component chondroitin sulfate A [4], [5] on host vascular endothelial cells to mediate iRBC adherence to vessel walls. The pattern of adherence is selective in that most sequestered infected iRBCs are found in the lungs, adipose tissue, brain and placenta [6]–[8]. The ability to sequester in the vasculature of certain tissues is thought to be advantageous to the parasite because it diminishes clearance of trophozoite- and schizont-containing iRBCs in the spleen and promotes factors that are beneficial for parasite growth [3], [9]. Sequestration in organs such as the lung is not without consequences, as large numbers of iRBCs in the lung have been hypothesized to precipitate events that result in lung injury [1].

*Plasmodium berghei* ANKA (PbA) infection in C57BL/6 mice recapitulates many characteristics of human *P. falciparum* infection including iRBC sequestration in the lungs [10] with pulmonary pathology that includes increased protein extravasation [11], [12], pulmonary edema [13] and hemorrhage [11], [13]. Work with synchronized PbA infections has demonstrated that schizont-containing RBCs selectively adhere to lung microvascular endothelial cells [10], [14], [15] and this interaction is dependent on capillary endothelial cell expression of CD36 [9], [10], [16]. Interestingly, the PbA genome does not contain any PfEMP1 orthologues. Recently, it has been demonstrated that the schizont membrane-associated cytoadherence protein (SMAC) is critical for PbA schizont-stage parasites to adhere to vascular endothelium via CD36 [9]. Thus, despite differences in the stages that sequester in the lungs between *P. falciparum* (schizonts, trophozoites and immature gametocytes) and PbA (schizonts only), both species adhere to the lung vascular endothelium via CD36.

The goal of the work presented here was to take advantage of the PbA-mouse model and an isolated perfused lung system to determine the role that CD36 interactions play in the changes to pulmonary vascular permeability observed during malaria infection. We found that PbA-infected CD36−/− mice were protected from the changes in fluid conductance observed in WT animals. Employing mouse lung microvascular endothelial cell cultures (MLMVEC), it was determined that CD36−/− endothelial cells were also protected from reactive oxygen species (ROS)-induced changes in barrier integrity. In addition, Fyn, a CD36-associated tyrosine kinase [17], [18], was shown to exhibit an altered intracellular distribution and activation status in CD36−/− LMVECs and to be critical for CD36-mediated increases in pulmonary endothelial cell fluid conductance during malaria. Our results suggest that CD36 signaling through Fyn tyrosine kinase plays a significant role in mediating the detrimental changes in pulmonary endothelial barrier function during malaria infection. In addition, the data point to the possibility that parasite- or host-derived ROS serve to enhance the CD36-mediated increases in paracellular fluid conductance during malaria. These findings suggest that targeting of the CD36-iRBC interaction in the lungs of patients at risk for pulmonary complications could reduce the severity of malaria-associated acute lung injury.

## Results

### CD36−/− mice are protected from *P. berghei*-induced lung injury

**Figure 1:**
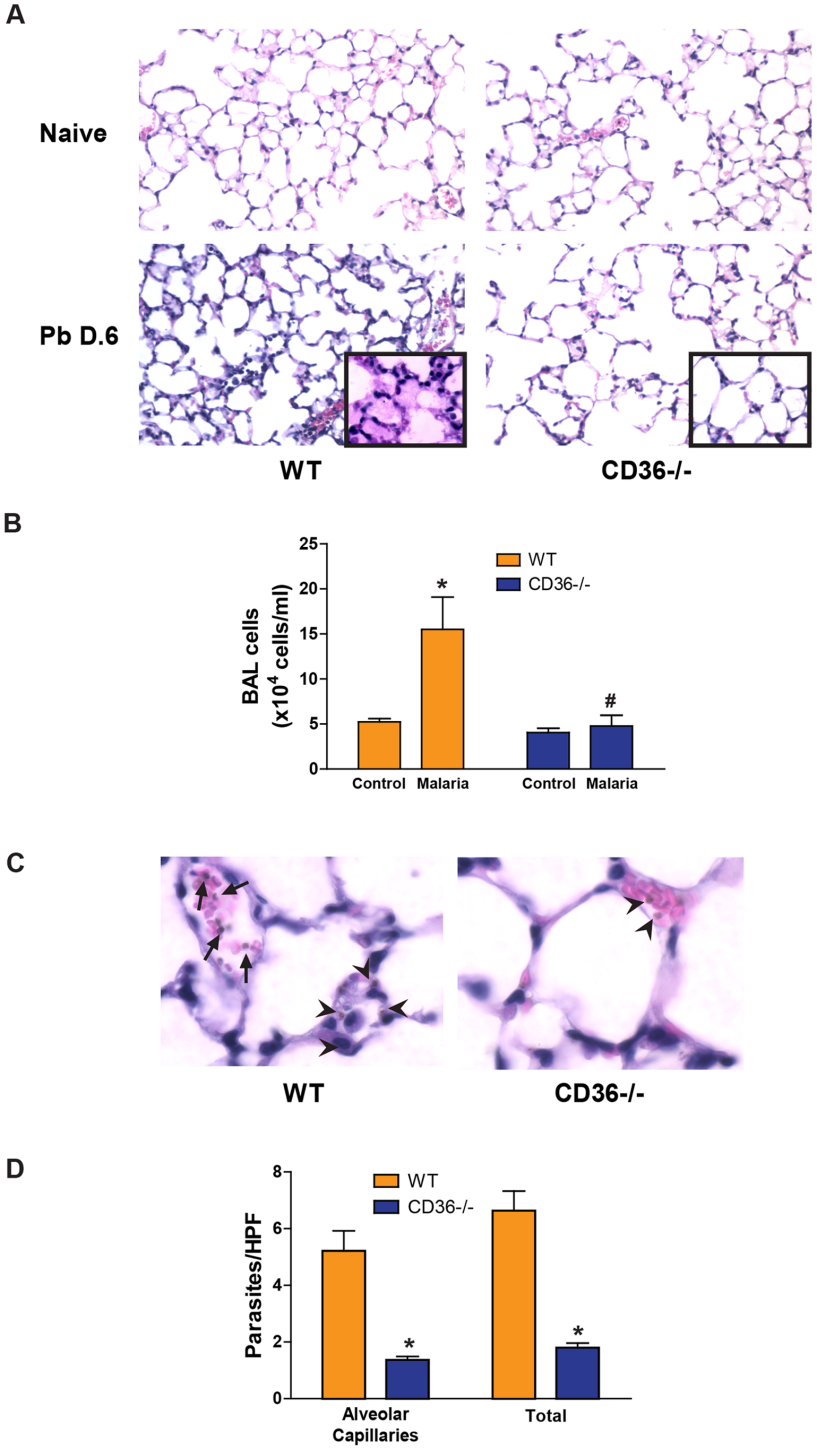
CD36−/− mice exhibit minimal malaria-induced lung pathology. **A**) Hematoxylin and eosin staining of histological lung sections from control and P. *berghei*-infected (day 6) WT and CD36−/− mice (10×). Inserts are representative higher-magnification fields (40×) of the septal thickening and edema seen in WT lungs compared to the lack of pathology in observed in the lungs from CD36−/− animals. **B**) Total number of cells isolated from the bronchoalveolar lavage fluid from uninfected and *P. berghei*-infected WT and CD36−/− mice. Data are means ± SE; n = 3–6. *P<0.05 vs. Control; #*P*<0.05 vs. WT malaria by ANOVA interaction and Kruskal Wallace testing. **C**) *P. berghei* within lung conduit vessel (arrows) and alveolar capillaries (arrowheads) of WT and CD36−/− mice (60×). **D**) The number of *P. berghei*/high power field (HPF) in the alveolar capillaries or in all vessels (Total) in the lungs of WT and CD36−/− mice. Data are means ± SE. n = 3 mice per group and 10 HPFs per lung section.*P<0.05 vs. WT by ANOVA.

There were no significant differences in the kinetics of blood parasitemia, weight loss and body temperature changes between PbA-infected WT and CD36−/− animals (Figure S1). However, at day 6 post-infection, WT animals had histologic evidence of lung injury characterized by thickened alveolar septae, patchy alveolar edema and increased inflammatory cell infiltration, while the lungs from CD36−/− animals were indistinguishable from the lungs of uninfected controls (Figure 1A and 1B). The heightened pathology observed in the WT lungs was associated with significantly greater numbers of parasites in the lungs by histology (Figures 1C and 1D) and by flow cytometry of perfused lungs (Figure S2). These results are consistent with previous reports that CD36 is a determinant of iRBC adherence in the lungs [10] and with the hypothesis that CD36-dependent iRBC-endothelial cell interactions contribute to pulmonary pathology during malaria.

### CD36 contributes to malaria-induced increases in lung paracellular fluid conductance

**Figure 2:**
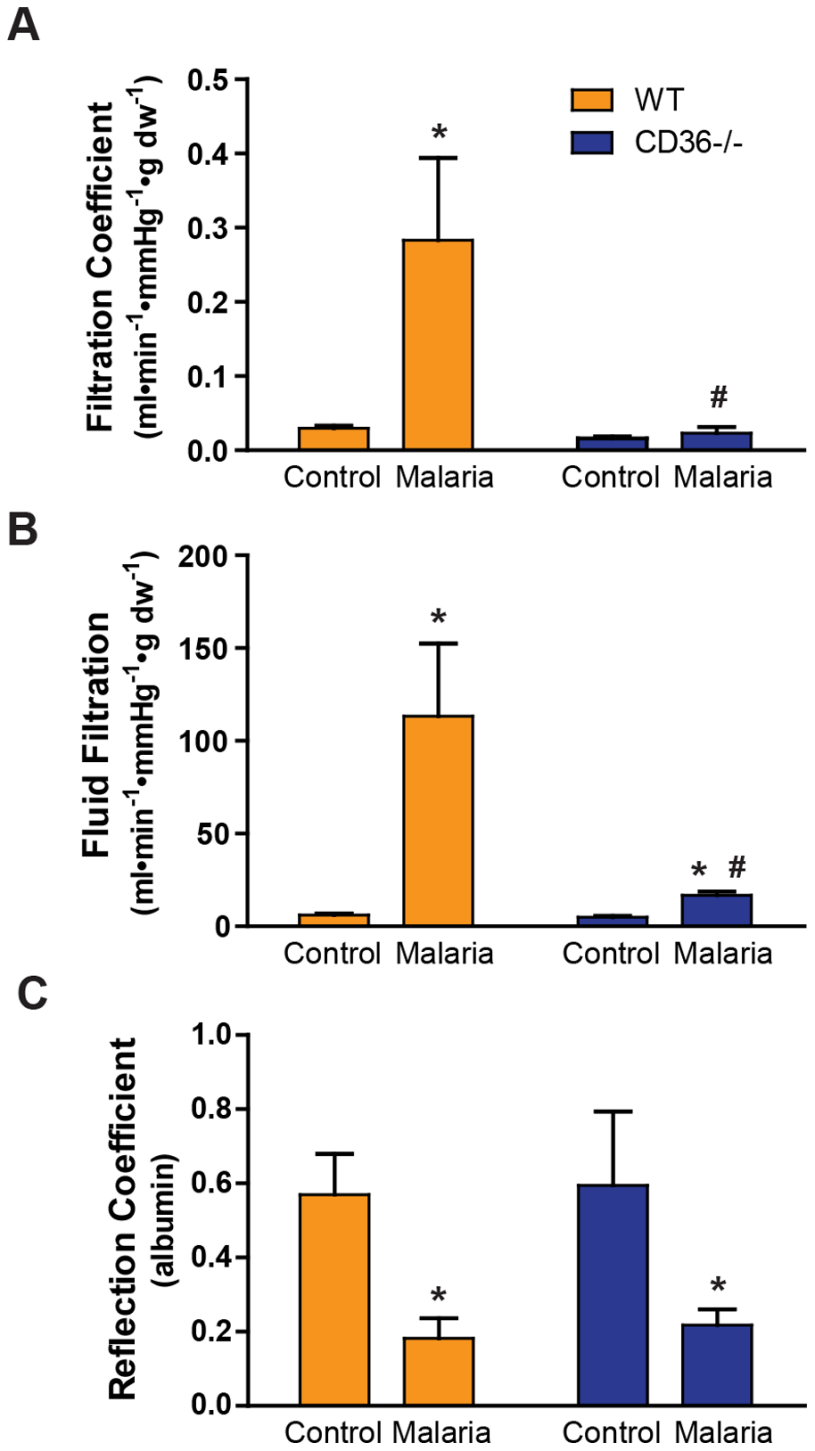
CD36−/− mice are protected from malaria-induced endothelial permeability. **A**) Effect of *P. berghei* infection on filtration coefficient in the lungs from WT and CD36−/− mice. Control mice received 10^6^ uninfected RBCs whereas malaria mice received 10^6^ *P. berghei*-iRBCs. Filtration coefficient was measured on day 6 post-injection as described in the Methods section. Data are means ± SE. n = 3–6; *P<0.02 vs. WT control; #*P*<0.05 vs. WT malaria by ANOVA interaction. **B**) Effect of *P. berghei* infection on the average fluid filtration in WT and CD36−/− mice. Average fluid filtration was determined from the change in perfusion circuit hematocrit and a time-weighted vascular pressure normalized to lung dry weight as described in the Methods section. Data are means ± SE. n = 3–6; *P<0.006 vs. corresponding control; ^#^ *P*<0.002 vs. WT malaria by ANOVA interaction. **C**) Effect of *P. berghei* infection on the albumin reflection coefficient (σ_alb_) in the lungs from WT and CD36−/− mice. σ_alb_ was measured by a modified filtered volume method as described in Methods. Data are means ± SE. n = 3–5; *P<0.005 vs. corresponding Control by ANOVA.

To characterize the effect of malaria infection on the pulmonary endothelial barrier and to determine the contribution of CD36, WT and CD36−/− mouse lungs were isolated and perfused with blood from donor mice after 6 days of malaria infection as described in the Methods. The filtration coefficient (Kf) and reflection coefficient for albumin (σ_alb)_ are components of the Starling equation, a calculation that is commonly employed to quantify paracellular fluid conductance and the effectiveness of the protein oncotic pressure gradient established by the endothelial barrier, respectively [19]. The estimated Kf by our methodology is a gravimetric measure with potential errors introduced by vascular volume change as well as circuit leakage or hemorrhage into the lung [20]. Therefore, we also calculated fluid filtration (Ff) which is the average rate of erythrocyte-free fluid volume entering the lung determined from the perfusate hematocrit change normalized to the time-weighted vascular pressure. In the isolated-perfused lung system, PbA infection of WT lungs increased the Kf and Ff >10-fold compared to controls (Figures 2A and 2B) indicating a marked increase in paracellular fluid conductance. In contrast, the lungs from PbA-infected CD36−/− mice had markedly attenuated changes in Kf and Ff indicating near-complete protection from malaria-induced paracellular hyperpermeability. Furthermore, we demonstrated a tight correlation between Kf and Ff (R = 0.91, P<0.0001, N = 27; data not shown) confirming the accuracy of our methods. Malaria infection also decreased σ_alb_ in the lungs from both PbA-infected WT and CD36−/− mice demonstrating a comparable reduction in σ_alb_ (Figure 2C), suggesting that the mechanism controlling the protein sieving properties of the paracellular junctions [19] was CD36-independent.

The observed CD36-associated effects on capillary endothelial barrier integrity could result from multiple mechanisms. Interaction with the iRBC may induce CD36-mediated signal transduction events in vascular endothelial cells [21] that cause alterations in permeability (see below). It is also possible that the tethered iRBC is a direct or indirect source of factors that enhance the hyperpermeabilty phenotype. For example, ROS are known to increase pulmonary microvascular permeability [22], [23]. ROS production has been associated with *P. falciparum* asexual development [24], [25] and implicated in the pulmonary pathology associated with severe malarial [26]–[28]. ROS can also be derived from resident or recruited mononuclear cells as they clear iRBCs from the lungs [29]. Here we investigated the possibility that PbA-infected RBCs were a source of ROS.

### Lung sequestered PbA-iRBCs are a source of reactive oxygen species

**Figure 3:**
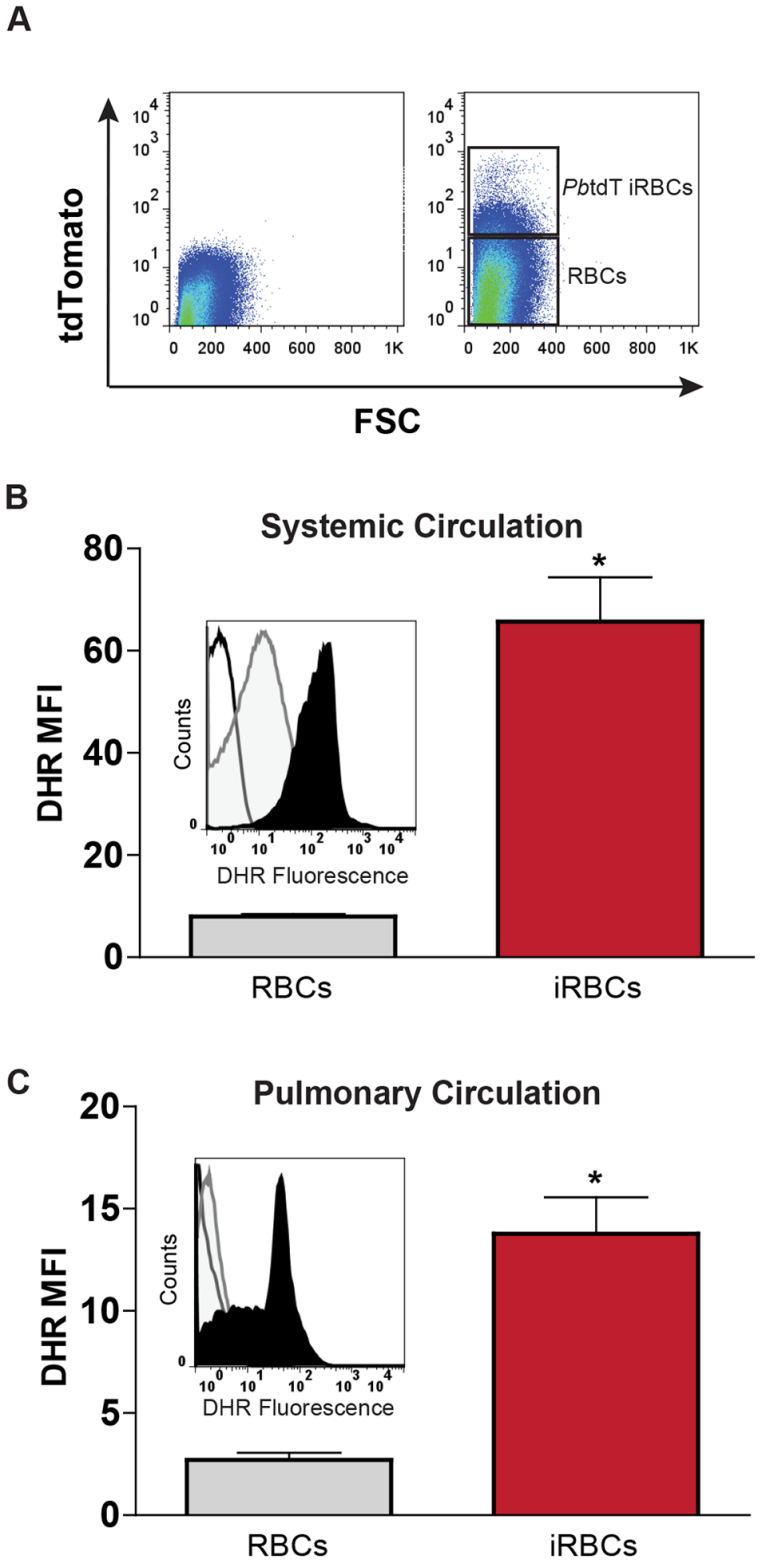
iRBCs generate reactive oxygen species (ROS) in the peripheral blood and pulmonary vasculature. **A**) Flow cytometric analysis of peripheral blood isolated from WT mice 6 days after infection with *P. berghei* ANKA (left panel) or the red fluorescent strain of *P. berghei* ANKA tdTomato (PbtdT) (right panel). The gating strategy used to identify iRBCs based on tdTomato fluorescence forward scatter (FSC) is defined in the right panel. **B**) ROS production in the peripheral blood of control or PbAtdT-infected mice. ROS production was measured by flow cytometry of cells isolated from control and PbAtdT-infected (day 6) animals and labeled with dihydrorhodamine (DHR) 123. Uninfected RBCs and iRBCs were separated based on the presence of the erythroid marker, ter119, and PbAtdT fluorescence. The data are expressed as mean fluorescence intensity (MFI) of the DHR 123. Data are means ± SE. n = 3–6; *P<0.001 vs. RBCs. Inset plot: histograms of DHR fluorescence of peripheral unstained control RBCs (white histogram), DHR-stained control RBCs (gray histogram), and DHR-stained iRBCs (black histogram). **C**) ROS production of control or PbAtdT iRBCs sequestered in the pulmonary vasculature. Lung-sequestered RBCs were isolated via dispersion of lung tissue after vascular perfusion with 10 ml of PBS to remove non-adherent cells. Lung-associated RBCs and iRBCs were stained and sorted for the presence of the erythroid marker, ter119, and PbAtdT fluorescence. Sorted cells were stained with DHR 123 for assessment of ROS production. The data are expressed as mean fluorescence intensity (MFI) of the DHR 123. Data are means ± SE. n = 3–6; *P<0.001 vs RBCs. Inset plot: histograms of DHR fluorescence of lung sequestered unstained control RBCs (white histogram), DHR-stained control RBCs (gray histogram), and DHR-stained iRBCs (black histogram).

It has been established that *P. falciparum*-infected RBCs generate ROS in the food vacuole and that the parasite-generated ROS egresses into the host cell compartment [24], [25]. To test whether *P. berghei* ANKA-infected RBCs also produce ROS, we employed dihydrorhodamine 123 (DHR), a cell-permeable fluorogenic molecule used in the detection of peroxide and peroxynitrite [30], [31]. To determine the level of ROS production by iRBCs in the peripheral circulation, blood was isolated from the tail veins of WT mice 6 days after infection with a tdTomato-expressing PbA strain (PbAtdT) and compared to uninfected controls (Figure 3A). PbA-infected erythrocytes (ter119^+^ tdTomato^+^) exhibited a significantly higher DHR mean fluorescence compared to uninfected RBCs (ter119^+^ tdTomato^−^) (Figure 3B) indicating that, similar to *P. falciparium* blood stage parasites, PbA also generates ROS.

In *P. falciparum*, erythrocytes containing trophozoites produce the highest level of ROS [25]. To assess the contribution of ROS production from schizont-infected RBCs sequestered within pulmonary vessels [15], lungs from PbAtdT-infected animals were extensively perfused to remove non-adherent cells prior to analyzing sequestered iRBCs for ROS levels by DHR fluorescence and flow cytometry. The iRBCs retained within the pulmonary vasculature were positive for ROS production (Figure 3C). These data demonstrate that iRBCs sequestered in the lung produce ROS that could contribute to increased vascular permeability. Of note, the level of ROS generated by the PbA schizont-infected RBCs in the lungs was lower than that detected in the iRBCs from the peripherial blood. Thus it appears that in PbA, like *P. falciparum*, there is a stage-dependent production of ROS that could serve to alter host responses.

### CD36 contributes to ROS-induced barrier changes

**Figure 4:**
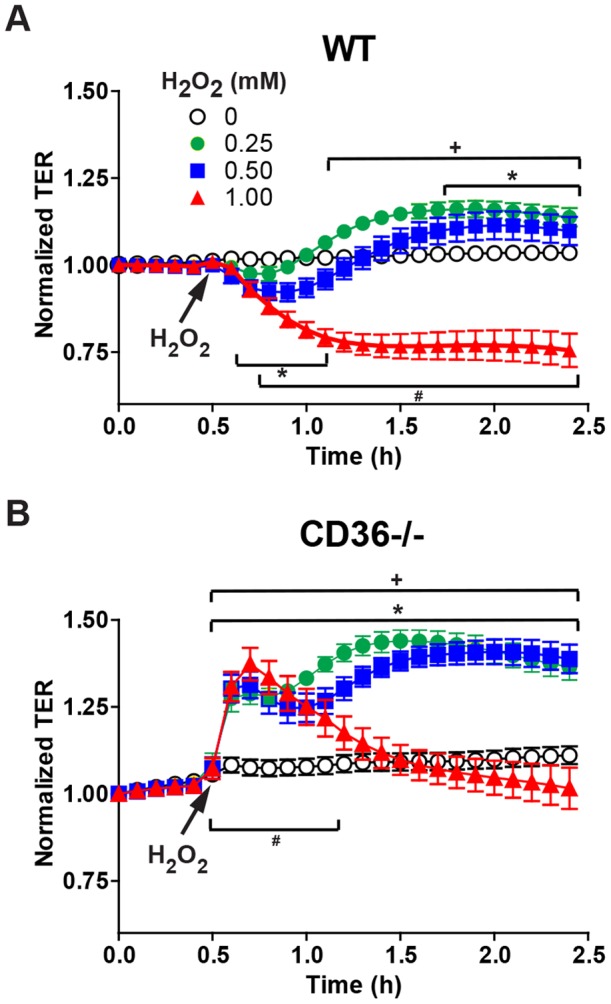
CD36−/− lung microvascular endothelial cells are protected from increases in H_2_O_2_-induced endothelial barrier permeability. Time course of trans-endothelial electrical resistance (TER) of mouse lung microvascular endothelial cell (MLMVEC) monolayers from WT (**A**) or CD36−/− (**B**) mice following exposure to increasing concentrations of H_2_O_2_ (0.25, 0.50 and 1.0 mM). All TER values were normalized to the TER measured 0.5 h before the addition of H_2_O_2_. Baseline TER for CD36−/− MLMVEC monolayers (1098±25 Ω; n = 63) was significantly greater (*P*<0.0001) than WT TER (902±25 Ω; n = 119). Data are means ± SE. n = 16–26; all symbols indicate *P*<0.00001 vs. diluent by 2-factor (H_2_O_2_ dose, time) ANOVA interaction; * refers to 0.25 mM H_2_O_2_, + refers to 0.5 mM H_2_O_2_ and # refers to 1 mM H_2_O_2_). The result of a 3-factor (genotype, H_2_O_2_ dose, time) ANOVA interaction F ratio was P<0.00001 for TER differing as a function of CD36 expression.

To determine if ROS could contribute to the observed changes in barrier function between WT and CD36−/− lungs, we employed a mouse lung microvascular endothelial monolayer (MLMVEC) system and measured the changes in transendothelial resistance (TER) before and after ROS challenge. TER is measured by passing a current across a cell monolayer and the overall impedance provides an indicator of integrity of cellular junctions. Basal TER was increased in CD36−/− MLMVECs compared to WT suggesting an inherently tighter barrier in the CD36-deficient MLMVEC (1098±25 vs. 902±26 Ω; N = 65–77; P<0.0001). In WT MLMVEC monolayers, H_2_O_2_ caused a dose-dependent decrease in TER during the first 30 minutes of exposure (Figure 4A). At 1 mM H_2_O_2_, TER continued to drop and remained low whereas the TER following 0.25 and 0.50 mM H_2_O_2_ returned to baseline and then recovered to levels that were significantly greater than that of untreated cells (Figure 4A). In contrast, all H_2_O_2_ concentrations resulted in a rapid increase in TER in the CD36−/− MLMVEC monolayers that remained elevated with 0.25 and 0.50 mM H_2_O_2_ (Figure 4B). Following 1 mM exposure, the increased resistance of the CD36−/− MLMVEC gradually returned to baseline. These data uncover a critical role for CD36 as a key mediator of ROS-induced endothelial cell barrier dysfunction.

**Figure 5:**
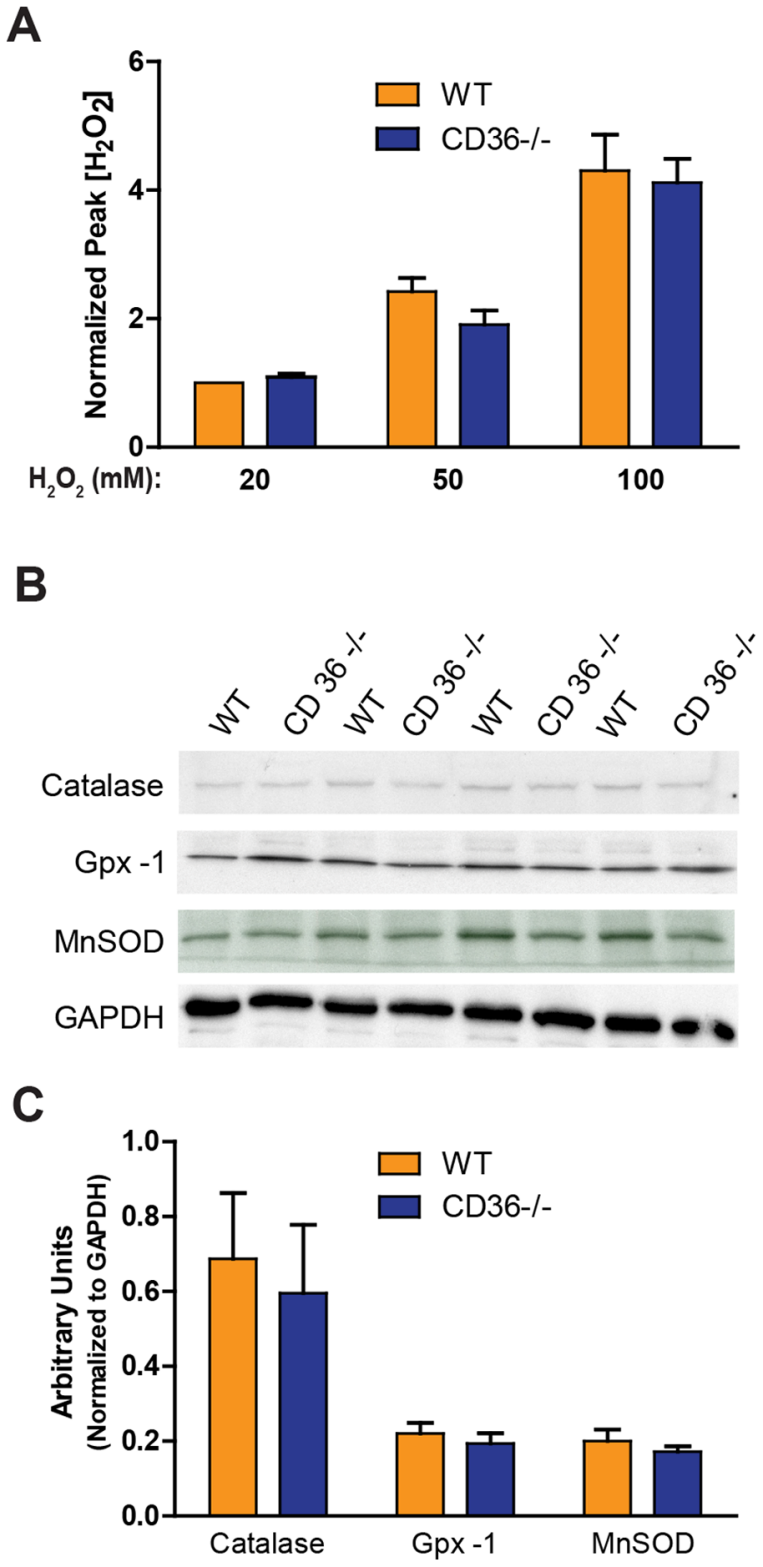
WT and CD36−/− MLMVECs scavenge exogenous H_2_O_2_ equally and display similar antioxidant enzyme expression. **A**) The dose-response relationship between increasing concentrations of H_2_O_2_ (20 μM, 50 μM, 100 μM) and the peak [H_2_O_2_] measured by electrode (see Methods section) from WT and CD36−/− MLMVECs. The peak [H_2_O_2_] values after 50 μM and 100 μM of H_2_O_2_ exposure were normalized to the 20 μM H_2_O_2_ control value. Data are means ± SE (n = 4–5). **B**) Immunostained Western blots of catalase, glutathione peroxidase 1 (Gpx-1) and manganese superoxide dismutase (MnSOD) from extracts of WT and CD36−/− MLMVECs. The blots show the results from four independent WT and four independent CD36−/− MLMVEC preparations. **C**) Quantitative analysis of the results of the immunostained Western blots of catalase, glutathione peroxidase 1 (Gpx-1) and manganese superoxide dismutase (MnSOD). Antioxidant enzyme expression was quantified by densitometry in arbitrary densitometry units and normalized to GAPDH levels. Data are means ± SE (n = 4–10).

To assess whether the observed protection of CD36−/− endothelial cells to ROS-mediated endothelial barrier dysfunction was due to enhanced antioxidant activity, we measured H_2_O_2_ scavenging and key antioxidant enzyme expression in WT and CD36−/− MLMVECs. Despite the marked differences in TER shown in Figure 4B, CD36−/− and WT MLMVECs scavenged a similar amount of extracellular H_2_O_2_ across a range of H_2_O_2_ concentrations (Figure 5A). Furthermore, Western blot analysis of three critical lung endothelial antioxidant enzymes, catalase, glutathione peroxidase-1 (Gpx-1), and manganese superoxide dismutase (MnSOD), revealed no differences between WT and CD36−/− MLMVECs (Figure 5B). These data suggest that the CD36−/− MLMVEC endothelial barrier protective properties were not mediated by enhanced antioxidant scavenging of H_2_O_2_.

### Fyn protein is dysregulated in CD36−/− MLMVECs

**Figure 6:**
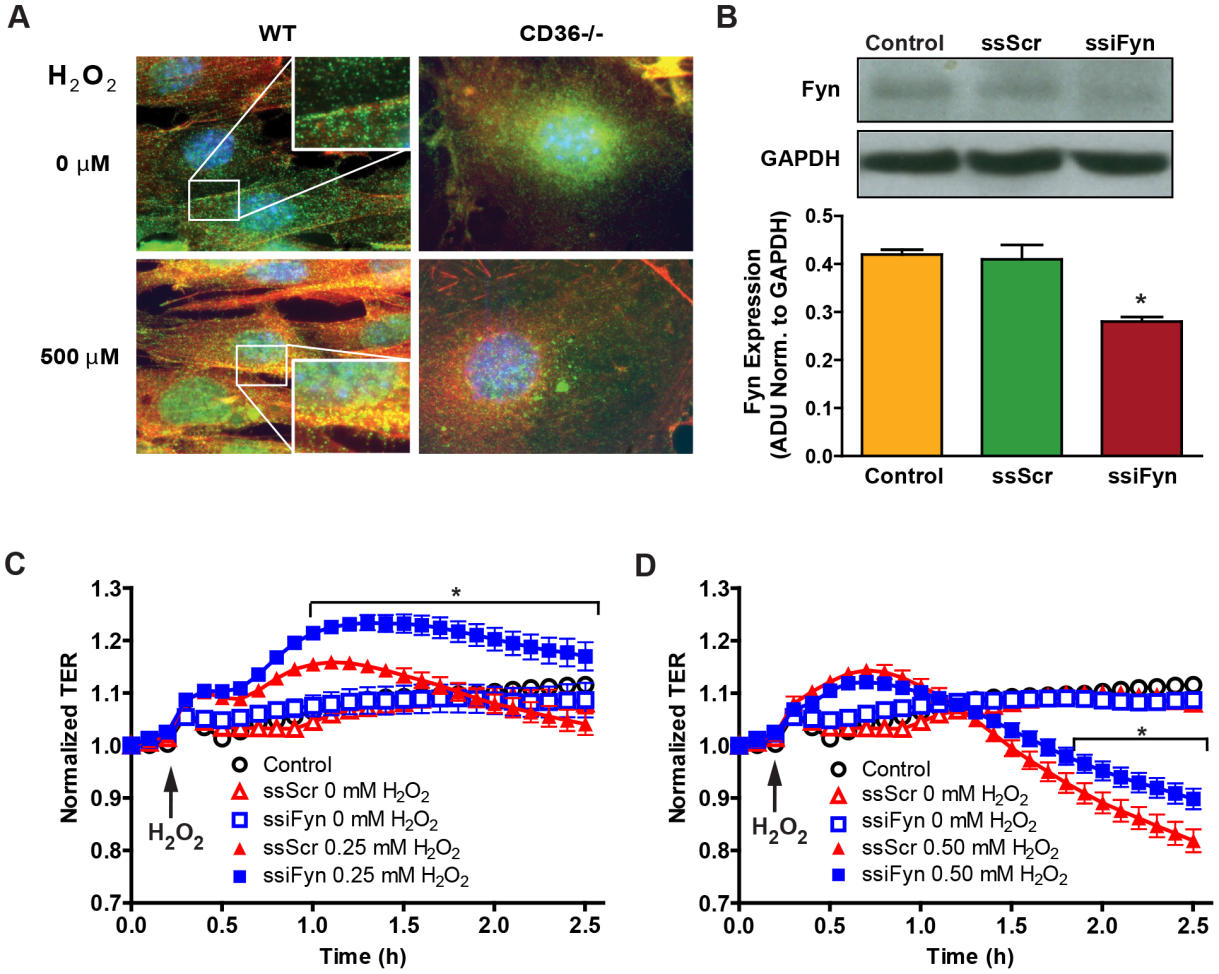
Fyn cellular compartmentalization and the impact of Fyn knockdown on H_2_O_2_-mediated endothelial barrier function. **A**) Effect of H_2_O_2_ exposure on WT (left panels) and CD36−/− (right panels) MLMVEC Fyn protein localization. MLMVECs stained for total Fyn (anti-Fyn, green), actin (Texas red-conjugated phalloidin) and nuclei (DAPI 4′, 6-diamidino-2-phenylindole, blue) without (top row) or with (bottom row) 60 min of exposure to 500 μm H_2_O_2_ (60×). Inserts show magnified images (100×). **B**) Efficiency of Fyn knockdown by sticky small interfering (ssi) RNA. Fyn expression in WT MLMVECs 48 hours after transfection with lipofectamine only (Control), scrambled (ssiScr) or Fyn (ssiFyn) ssiRNA was assessed by Western blot with quantification by densitometry. The data are expressed in arbitrary absorption units before normalization to GAPDH levels. Data are means ± SE. n = 3; *P<0.05 vs. ssScr. **C**, **D**) Time series of normalized trans-endothelial electrical resistance (TER) in WT MLMVEC monolayers following exposure to (**C**) 0.25 mM or (**D**) 0.50 mM H_2_O_2_. Open symbols include Lipofectamine only, ssiScr and ssiFyn all in the absence of H_2_O_2_. Closed symbols include ssiScr-transfected or ssiFyn-transfected MLMVEC monolayers exposed to H_2_O_2_. Data are means ± SE. n = 3–5; *P<0.002 vs. ssScr by ANOVA interaction.

Because of a known association of CD36 with Fyn kinase in dermal microvascular endothelial cells [17] and CD36-dependent recruitment of activated Fyn [18], the distribution of Fyn in WT and CD36−/− MLMVEC monolayers was determined (Figure 6A). In WT cells at baseline (top left), Fyn was present throughout the cytoplasm in a focal adhesion-like pattern, but in CD36−/− cells (top right), Fyn was predominantly peri-nuclear with no concentration at focal adhesions or cell-cell interfaces. After H_2_O_2_ challenge of WT MLMVECs, Fyn became strongly co-localized with actin stress fibers. H_2_O_2_ challenge of CD36−/− MLMVEC monolayers resulted in no significant change in the distribution of Fyn, which retained a diffuse peri-nuclear pattern. Thus, CD36 deficiency was accompanied by a notable alteration in the cellular distribution of Fyn in LMVECs suggesting that altered Fyn-mediated signaling may play a role in the observed changes in barrier function in CD36−/− lungs.

### Fyn knockdown protects MLMVEC from changes in paracellular permeability

To determine if disruption of Fyn-mediated signaling could recapitulate the barrier function phenotype of CD36−/− monolayers, a sticky short interfering RNA (ssiRNA) approach was employed to reduce the levels of Fyn in WT MLMVEC monolayers prior to H_2_O_2_ challenge. Under the conditions used here, ssiFyn decreased Fyn expression by ∼33% (Figure 6B). This level of Fyn knockdown had no demonstrable effect on baseline TER (data not shown), but it did significantly increase the TER of monolayers treated with 0.25 and 0.50 mM H_2_O_2_ when compared to their respective scrambled ssiRNA (ssiScr) control sequence groups (Figures 6C and 6D). At the 1 mM H_2_O_2_ challenge dose, the TER values for the ssiFyn-treated cells were not significantly different from ssScr controls (data not shown). The protective phenotype observed as a result of this partial depletion of Fyn is consistent with the idea that CD36 signals via Fyn to modulate paracellular permeability.

### CD36 is critical for Fyn signaling in the lungs following PbA infection

**Figure 7:**
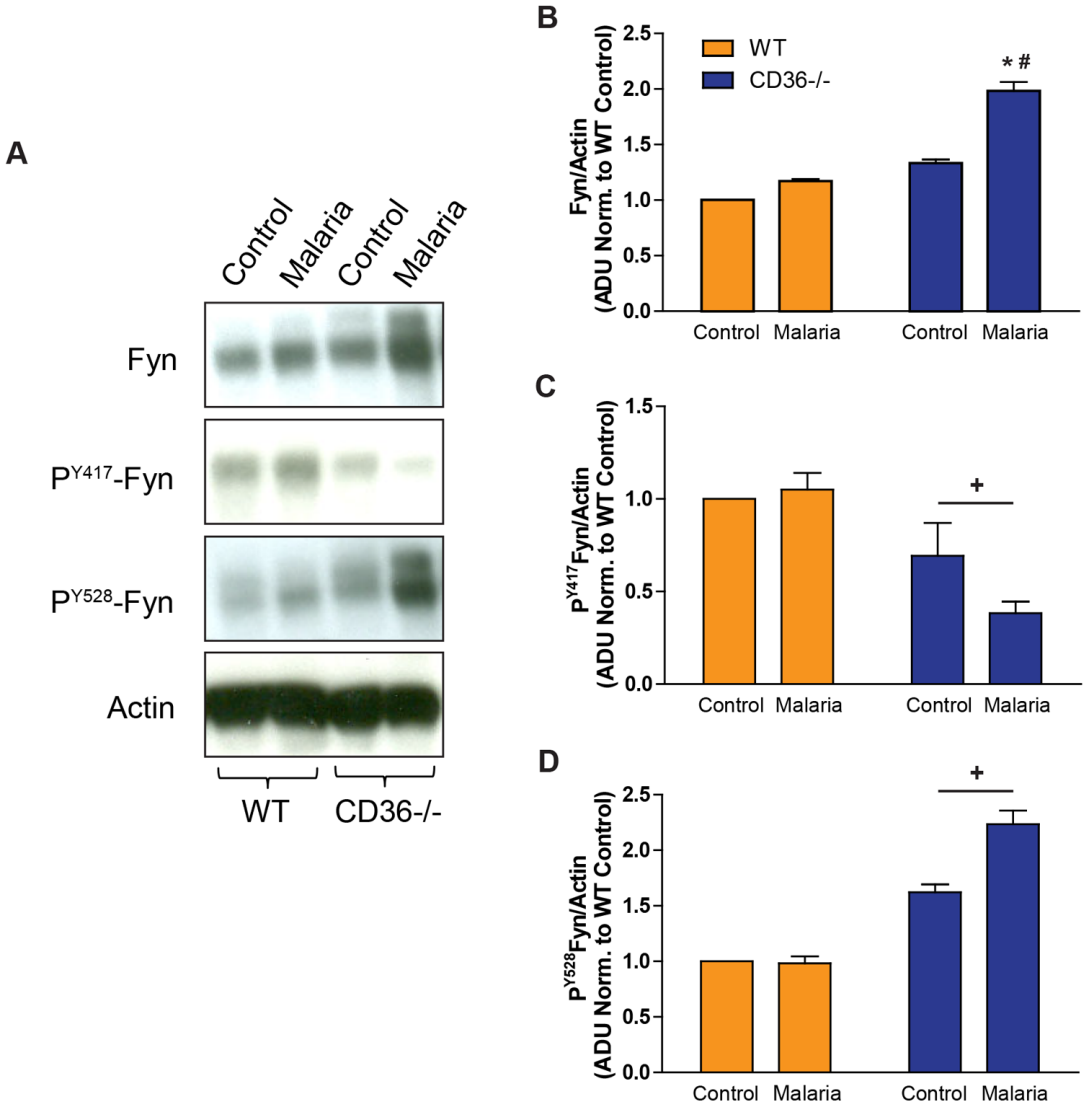
Altered Fyn protein level and activation in the lungs of malaria-infected CD36−/− mice. **A)** Immunostained Western blots measuring total Fyn, P^Y417^-Fyn (activated form) and P^Y528^-Fyn (inactivated form) in lung homogenates from control and malaria-infected (day 6) WT and CD36−/− mice. **B–D**) Densitometry quantification of total Fyn (**B**), P^Y417^-Fyn (**C**) and P^Y528^-Fyn (**D**) normalized to actin expression. Data are expressed in arbitrary densitometry units (ADU) normalized to WT control values and represent the mean ± SE. n = 3–5; *P<0.05 vs. corresponding control; #, *P*<0.05 vs. WT malaria by ANOVA interaction; +,P<0.03 vs. WT values by ANOVA main factor effect.

Based on evidence of Fyn dysregulation in CD36−/− MLMVECs, we examined the production of total, active (P^Y417^-Fyn) and inactive Fyn (P^Y528^-Fyn) in lung homogenates from control or day 6 post-PbA infected WT and CD36−/− mice. Total Fyn protein levels were unchanged in malaria-infected WT lungs compared to control, but significantly increased in PbA-infected CD36−/− lungs compared to both control lungs and infected WT lungs (Figures 7A and B). The increased Fyn levels in CD36−/− lungs were largely inactive P^Y528^-Fyn that was significantly increased compared to WT mice. There was a corresponding and significant decrease in the active P^Y417^-Fyn in CD36−/− mice compared to WT levels (Figures 7A, C and D).

**Figure 8:**
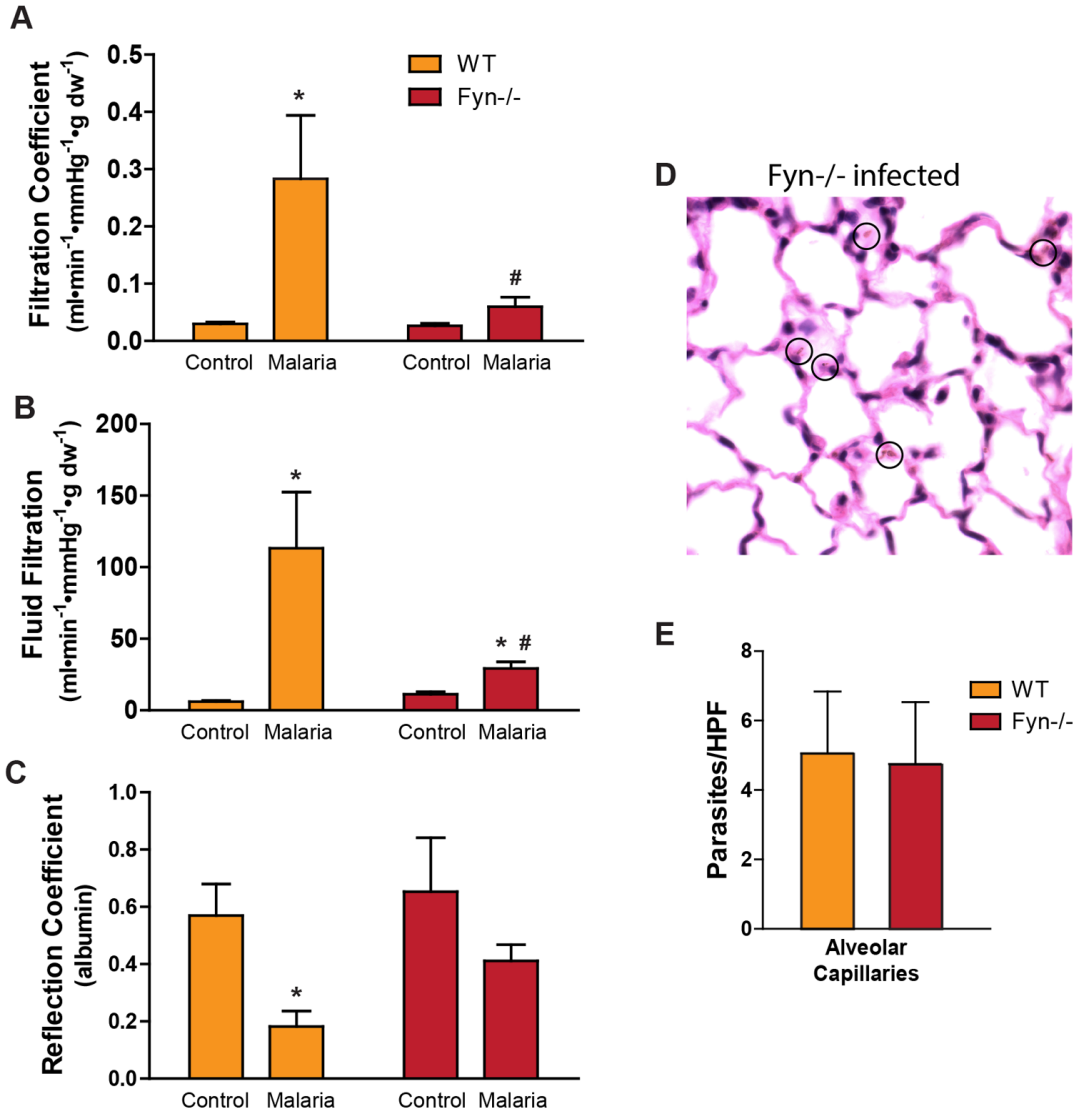
Fyn−/− mice are protected from malaria-induced increases in pulmonary endothelial barrier permeability. **A**) Effect of *P. berghei* infection on filtration coefficient in the lungs from WT and Fyn−/− mice. Filtration coefficient was measured via isolated lung perfusion on day 6 post-infection as described in the Methods. Data are means ± SE. n = 3–6; *P<0.02 vs. corresponding control; #*P*<0.05 vs. WT malaria by ANOVA interaction. **B**) Effect of *P. berghei* infection on the average fluid filtration in WT and Fyn−/− mice. Average fluid filtration was determined from the change in perfusion circuit hematocrit and a time-weighted vascular pressure normalized to lung dry weight as described in the Methods. Data are means ± SE. n = 3–6; *P<0.006 vs. corresponding control; ^#^ *P*<0.002 vs. WT malaria by ANOVA interaction. **C**) Effect of *P. berghei* infection on the albumin reflection coefficient (σ_alb_) in the lungs from WT and Fyn−/− mice. σ_alb_ was measured by a modified filtered volume method as described in Methods. Data are means ± SE. n = 3–5; *P<0.01 vs. corresponding control by ANOVA main factor (malaria infection) effect. **D**) Hematoxylin and eosin staining of histological lung sections from P. *berghei*-infected (day 6) Fyn−/− mice (60×). Circles identify *P. berghei* ANKA within alveolar capillaries. **E**) The number of *P. berghei*/high power field (HPF) in the alveolar capillaries in the lungs of WT and Fyn−/− mice. Data are means ± SE. n = 3 mice per group and 10 HPFs per lung section.

The role Fyn plays in malaria-induced endothelial barrier function was explored further using Fyn−/− mice. PbA-infected Fyn−/− animals achieved parasitemia and exhibited morbidity/mortality characteristics that were comparable to WT and CD36−/− mice (Figure S1). The lungs from uninfected control Fyn−/− mice had similar baseline endothelial barrier parameters to WT and CD36−/−− controls (Figures 8A–8C). Malaria infection in Fyn−/− mice resulted in changes in Kf and Ff (Figures 8A–8C) that were similar to that recorded for CD36−/− mice (Figure 2). Malaria infection decreased σ_alb_ in both WT and Fyn−/− lungs but the decrease in Fyn−/− did not reach statistical significance. Of note, histological analysis of malaria-infected Fyn −/− lungs revealed a lack of edema and septal thickening comparable to that observed in CD36−/− lungs (Figures 1A, 8D) despite having parasite numbers in the pulmonary vasculature that were comparable to that observed in the lungs from WT animals (Figure 8E). These data suggest that iRBC-CD36-Fyn interactions are a major regulator of lung barrier function during malaria infection. In addition, these data indicate that, in the absence of the ability of CD36 to properly signal, ROS produced by parasites have a minimal impact on paracellular water conductance.

## Discussion

It is well established that during severe malaria infection in humans, parasite-containing erythrocytes adhere to pulmonary capillary endothelium [2], [32] and that this sequestration is associated with ARDS in up to 25% of adults. *P. flaciparium*-infected RBCs appear to use distributive interactions with CD36, ICAM1, PCAM-1 and chondroitin sulfate A to adhere to vascular endothelial cells [4], [5]. In murine models of human malaria, CD36 on vascular endothelial cells is the major binding partner for iRBCs [10], [33], [34]. The high level of CD36 expression in the lungs [35] results in the sequestration of large numbers of iRBCs with an attendant development of pulmonary inflammation and injury [11]. A prominent feature of the lung pathology in humans and mice is pulmonary edema, suggesting that CD36-iRBC interactions could contribute directly or indirectly to alterations in endothelial cell barrier integrity. The results reported here support a mechanism in which iRBC interactions with CD36 initiates Fyn-mediated signaling that leads to an alteration in endothelial cell barrier function and pulmonary edema. In addition, our data identify parasite- or host-derived ROS as a possible contributor to this response.

*P. berghei* infection in WT animals resulted in >10-fold increase in paracellular fluid conductance (Kf and Ff; Figures 2A, B) and a marked decrease in the reflection coefficient (σ_alb_) indicating failure of the endothelial mechanisms that exclude water and plasma proteins from the lungs. In striking contrast, the lungs of malaria-infected CD36−/− animals demonstrated significantly decreased water conductance when compared to WT mice, consistent with the observed lack of histological evidence of pulmonary edema after malaria challenge (Figure 1). These results support a mechanistic link between CD36-mediated sequestration of iRBCs in the lungs and malaria-induced pulmonary paracellular hyperpermeability.

Interestingly, CD36 deficiency did not attenuate the malaria-induced decrease in the reflection coefficient (σ_alb_). Recent evidence suggests that the oncotic gradient is largely determined by the sieving properties of the glycocalyx, a complex network of surface glycopoproteins that covers the luminal surface of vascular endothelial cells and extends into the intercellular clefts [19], [36]. Thus, it is likely that the low σ_alb_ from malaria infection indicates loss of glycocalyx integrity. This putative loss of the glycocalyx appears to be a CD36-independent event, perhaps due to the activation of an endogenous heparanase by circulating cytokines [37].

It is possible that the lack of change in vascular permeability observed in the CD36−/− lungs during infection merely reflects a lack of signaling due to the significantly reduced number of iRBCs binding to the pulmonary vascular endothelium in the absence of CD36 (Figure 1D). While we acknowledge this possibility, the nearly identical permeability phenotype of Fyn−/− lungs, where CD36-dependent signaling capacity is presumably compromised but the vascular endothelium binds near WT levels of iRBCs, argues in favor of the interpretation that CD36-Fyn signaling is a key regulator of barrier integrity during infection.

We have demonstrated previously that ROS can increase pulmonary endothelial paracellular fluid permeability [23], [38]. One possible source of ROS in malaria is the iRBC. The degradation of hemoglobin in the food vacuole of a *P. falciparum* iRBC produces free heme and ROS such as H_2_O_2_ and hydroxyl radicals [24]. We report here for the first time that both circulating and lung-sequestered *P. berghei*-infected erythrocytes also generate ROS (Figures 3B and 3C). It is possible that the ROS generated from immobilized schizont-containing RBCs (or ROS released from non-sequestered iRBC as they pass through the lungs) contributes to the disruption of lung barrier function. However, the result that Fyn−/− mice are protected from malaria-induced paracellular hyperpermeability despite the presence of adhering iRBCs suggests that the contribution of parasite-derived ROS to the disruption of barrier function may be less important. It remains to be determined the extent to which ROS produced from iRBCs directly contributes to the malaria-induced increases in vascular permeability.

There are additional iRBC-related factors that may contribute to the malaria-induced increase in fluid permeability. Macrophages have been shown to be important in phagocytizing malaria-infected RBCs in the spleen [39] and are implicated in controlling the parasite load in the lungs [40]. It is possible that parasite-activated macrophages or other inflammatory cells also produce ROS to cause parasite killing [41]. In addition, Gillrie et al [42], [43] have shown that free merozoite proteins have an adverse effect on microvascular endothelial permeability *in vitro*, suggesting that rupturing of iRBCs in lung capillaries may play a role. Further work is required to define the relative roles of CD36 ligation, ROS and other parasite-derived factors in the adverse effects on the pulmonary endothelial barrier.

The *in vitro* data in MLMVEC monolayers and the *in vivo* results in malaria-infected lungs implicate Fyn kinase as a critical downstream mediator of CD36-dependent lung endothelial dysfunction. Fyn is known to regulate endothelial barrier function [44] and to co-localize with the cytoplasmic domain of CD36 in microvascular endothelial cells [17], [18]. We found that Fyn was recruited to and co-localized with the actin cytoskeleton in WT MLMVEC treated with H_2_O_2_ (Figure 6A) similar to activated Src [45]. In contrast, CD36−/− MLMVECs maintained a perinuclear localization and failed to recruit Fyn to the actin cytoskeleton following H_2_O_2_ challenge (Figure 6A). Further evidence of Fyn dysregulation in the absence of CD36 was obtained from malaria-infected CD36−/− lungs where it was demonstrated that the levels and activation status of Fyn were altered. The activity of Fyn is controlled by dephosphorylation of Tyr^528^ which triggers activation by autophosphorylation of Tyr^417^ [46]. We found that Fyn protein levels were significantly increased in CD36−/− compared to WT lungs, but the increased Fyn was largely in the inactive form (Figures 7A–D). Although the mechanism for this is unknown, we speculate that a physical association of Fyn with CD36 may be required for Fyn activation perhaps because CD36 facilitates the interaction of Fyn with the phosphatase responsible for dephosphorylating Tyr^528^.

Fyn−/− mice were markedly protected from the PbA-induced lung endothelial barrier dysfunction (Figure 8). The nearly identical phenotype in the CD36−/− and Fyn−/− mice supports the hypothesis that CD36 and Fyn are functionally linked in the mechanism that regulates pulmonary endothelial barrier integrity in malaria. However, because Fyn has also been shown to be important in T cell function [47]–[50], we cannot exclude the possibility that a component of the phenotype observed *in vivo* in the Fyn−/− lungs was due to the effects of an altered level of immune responsiveness. Additional studies are planned to determine if there are significant changes in cytokine response in Fyn−/− animals and to what extent these changes might contribute to the endothelial barrier protection.

In conclusion, this study is the first to show the critical roles that CD36 and the tyrosine kinase Fyn play as mediators of the increased pulmonary endothelial permeability found in malaria-infected mice. In addition, these data suggest that CD36 is necessary for the appropriate cellular compartmentalization and activation of Fyn and that infection–induced ROS may serve as an enhancing factor in the mechanism that results in pulmonary edema. Although additional experiments are necessary to fully elucidate these mechanisms, our data identify new potential targets for future treatments of malaria-induced lung injury.

## Materials and Methods

### Ethics Statement

All animal protocols and procedures were approved by the Johns Hopkins Animal Care and Use Committee (JHACUC). The JHACUC and the University are fully in compliance with and follow all applicable guidelines of the Animal Welfare Act regulations and Public Health Service Policy. In addition, Johns Hopkins maintains accreditation by the Association for the Assessment and Accreditation of Laboratory Animal Care International.

### Animals

Wild-type (WT) male C57BL/6J mice and Fyn −/− mice on a C57BL/6 background were purchased from the Jackson Laboratories (Bar Harbor, ME). CD36−/− mice on a C57BL/6 background were obtained from Dr. Maria Febbraio (Cleveland Clinic Foundation) [51]. Both original congenic knockout mouse strains were backcrossed >10 times into C57BL/6 mice. Mice were maintained in a pathogen-free environment by the Johns Hopkins Division of Comparative Medicine.

### Parasites and infection

*Plasmodium berghei* ANKA (PbA) was obtained from The Malaria Research and Reference Reagent Resource Center (MR4, ATCC, Manassas, VA; P. berghei ANKA cl15cy1, catalog number MRA-871). Transgenic *P. berghei* ANKA expressing the red fluorescent protein tdTomato (PbAtdT) was obtained from Dr. Volker T. Heussler (Bernard Nocht Institute, Hamburg, Germany) [52]. Parasite infectivity was standardized by sequential passage of a frozen parasite stock through three C57BL/6J donor mice. Asynchronous infections were established in test C57BL/6J mice via intra-peritoneal (IP) injection of 10^6^ iRBCs obtained from the blood of the third donor. Control animals received 10^6^ uninfected RBCs IP. Starting on day 3 post-infection, parasitemia was monitored by assessing Giemsa-stained thin blood smears. Infection morbidity was assessed by measuring changes in body weight (CS200 digital balance, Ohaus) and core temperature (TH-5 thermometer, Physitemp).

### Lung histology and BAL

On day 6 post-infection, mice were anesthetized with IP administration of ketamine (150 mg/kg) and acetylpromazine (15 mg/kg). The trachea was intubated and the mouse exsanguinated from the femoral artery. Following sternotomy, the pulmonary vasculature was flushed with 2 ml of PBS through the right ventricle. Right lung BAL cell counts and differentials were determined as previously described [53]. The left lung was instilled with 650 µl of 1% agarose in 4% paraformaldehyde at 37°C via the tracheal catheter and suspended in 4% paraformaldehyde for 48 hours. The lungs were embedded in paraffin [54] and 10 µm sections were obtained from four levels. Hematoxylin and eosin-stained sections were examined using a Nikon Eclipse E800 microscope (Nikon Instruments, Melville, NY) and images were acquired using SPOT RT charge-coupled device imager and software (Spot Imaging Solutions, Sterling Heights, MI).

### Isolated, perfused mouse lung

The isolated lung perfusion was performed as previously described [55]. After anesthesia with ketamine and acetylpromazine, a tracheostomy was performed and room air ventilation initiated with 6 ml/kg tidal volume, 120 breaths/minute and 3 mmHg positive end-expiratory pressure (MiniVent Mouse Ventilator Type 845; Harvard Apparatus). After sternotomy, cannulas were secured in the pulmonary artery and left heart. The inspired gas was changed to 21% O_2_–5% CO_2_. The lungs were perfused (2.5 ml/minute) with warmed (37°C) blood from day 6 infected or uninfected donor mice diluted to 15–20% hematocrit with Brinster's BMOC-3 media (Invitrogen 11126034) containing 3% Bovine Serum Albumin Fraction V (Sigma).

### Pulmonary vascular permeability

The filtration coefficient (Kf) was measured as previously described [55]. Briefly, left atrial pressure was increased from 10 to 40 mmHg in 5 mmHg increments at 15-minute intervals. Perfusion was stopped when 40 mmHg was reached or if tracheal edema occurred. The Kf was estimated from the slope of the relationship between the change in reservoir weight over the final 5 minutes at each pressure and the mean pulmonary vascular pressure normalized to lung dry weight. To allow a simultaneous measurement of average fluid filtration (Ff) and the reflection coefficient for albumin (σ_alb_), perfusate samples were collected for measurement of hematocrit (Hct) and albumin concentration. Ff is an estimate of the average rate of RBC-free fluid volume that left the perfusate during the increase in pressure. Total filtered fluid volume was calculated from the starting circuit volume and the ΔHct. The filtered fluid volume was then divided by total filtration time and a time-weighted vascular pressure normalized to lung dry weight. Ff is unaffected by vascular volume change, circuit leakage or lung hemorrhage. The σ_alb_ was calculated via the filtered volumes technique as previously described [56]. The right lung was used for dry weight determination and the left lung was snap-frozen and stored at −80°C.

### Reactive oxygen species

Flow cytometry was employed to measure ROS production by iRBCs. A strain of *P. berghei* ANKA that expresses the red fluorescent protein tdTomato (PbAtdT) under the constitutively-expressed eukaryotic elongation factor 1 alpha promoter was used [52]. Mice were infected with 1 million PbAtdT RBCs and peripheral blood was collected 6 days post-infection directly into FACS Buffer (1X PBS, 5% heat-inactivated FBS) and stored at 25°C in the dark prior to analysis. To isolate iRBC sequestered in the pulmonary vasculature, on day-6 post-infection mice were anaesthetized with 700 μl of 2, 2, 2-tribromoethanol (Sigma, St. Louis, MO) and the lungs were perfused with 10 ml PBS via the right ventricle to remove nonadherent iRBCs. The lungs were excised, suspended in 5 ml RPMI 1640 containing 1 mg/ml collagenase type II (Gibco) and 30 μg/ml DNase I (Roche) and minced. Suspensions were passed through a 70-μm mesh nylon cell strainer (BD Falcon) and the resulting cells pelleted at 1500 RPM (4°C) and washed twice in FACS Buffer. Pellets were then suspended in 200 μl FACS buffer containing Fc Block (BD Biosciences, San Jose, CA) and incubated on ice for 5 minutes. Intracellular ROS was measured using dihydrorhodamine 123 (DHR) (Sigma) and erythrocytes were stained with APC-conjugated anti-mouse ter119 (eBioscience). Data were acquired on a FACSCalibur flow cytometer using CellQuest software (BD Biosciences). Data were analyzed using FloJo software (Tree Star).

### Lung microvascular endothelial cell isolation and culture

Mouse lung microvascular endothelial cells (MLMVECs) were isolated and purified as previously described [57]. Briefly, WT C57BL/6 and CD36−/− mice were sacrificed by cervical dislocation. Peripheral lung was rinsed with DMEM (Invitrogen), minced, and digested in 1 ml of collagenase (1 mg/ml; Sigma) at 37°C for 20 minutes. The digest was filtered through sterile mesh and centrifuged (400 g for 7 minutes). The pellet was suspended in DMEM supplemented with 20% FBS, 150 μg/ml ECGS, 100 μg/ml penicillin/streptomycin, 0.25 μg/ml amphotericin B, and placed in a 0.1% gelatin-coated T-25 flask. After reaching confluence, the cells were stained overnight with acetylated LDL/Alexa Fluor 488 conjugate (Molecular Probes/Invitrogen L23380), an endothelial cell marker, and sorted into a purified endothelial population using a FACS ARIA (Becton Dickinson). Endothelial phenotype was confirmed by observing typical cobblestone morphology and immunostaining for platelet endothelial cell adhesion molecule and von Willebrand factor. All experiments were performed with cells between passages 2 and 10.

### Transendothelial electrical resistance (TER)

Measurement of TER was performed with ECIS Z Theta (Applied BioPhysics,). Mouse lung microvascular cells from WT C57BL/6 male mice and from CD36−/− male mice were grown to confluence on 8w10e+ gold electrode plates in low serum media (DMEM Low Glucose (Gibco) containing 1% FBS Heat Inactivated; Sigma). Current was applied across the electrodes with amplitude of 1 V in series with a resistance of 1 MΩ to approximate a constant current source (∼1 µA). The in-phase and out-of-phase voltages between the electrodes were monitored and converted to TER. Baseline resistance for each cell preparation was measured just prior to experimental manipulation. The effect of H_2_O_2_ at increasing concentrations (0.25 mM, 0.50 mM, or 1.0 mM) on TER was assessed over 2.5 hours.

### MLMVEC H_2_O_2_ scavenging assessment

Real-time H_2_O_2_ concentrations were measured with an H_2_O_2_ electrode system (Apollo 4000 Free Radical Analyzer; World Precision Instruments) as previously described [58]. Briefly, an ISO-HPO-2 electrode was mounted in a multiport water-jacketed sample chamber (NOCHM-4 Four-Port Closed Chamber, World Precision Instruments). WT and CD36−/− MLMVECs were washed twice with PBS, collected with trypsin, centrifuged, and suspended in 2 ml of serum-free DMEM. The cell suspension was warmed to 37°C and stirred before H_2_O_2_ was added to achieve a specified concentration. The H_2_O_2_ signal was allowed to completely decay before the next concentration of H_2_O_2_ was added.

### Immunofluorescence staining

MLMVECs were grown on gelatinized glass dishes before being exposed to hydrogen peroxide. The cells were fixed in 4% paraformaldehyde for 10 minutes, washed three times with Tris-buffered saline solution containing 0.1% Tween 20 (TBS-T, Sigma), permeabilized with 0.2% Triton X-100 in PBS for 5 minutes, and blocked with 2% BSA in PBS for 30 minutes. MLMVECs were then stained with mouse anti-mouse Fyn (1∶200, Santa Cruz) for 1 hour followed by Alexa 488 goat anti-mouse IgG (1∶200, Molecular Probes) for 1 hour at room temperature. Actin filaments were visualized by staining cells with Texas red-conjugated phalloidin (Molecular Probe) in similar fashion. After three washes with TBS-T, the coverslips were mounted with a SlowFade® AntiFade Kit (Molecular Probes) and analyzed using an Olympus IX51 inverted fluorescent microscope (60× oil objective) and a Cooke digital camera.

### ssiRNA Fyn knockdown

The siRNA sequence targeting mouse Fyn was generated using an siRNA design tool (www.ambion.com). Fyn-specific target sequences were aligned to the mouse genome database in a BLAST search to eliminate sequences with significant homology to other mouse genes. Two mRNA targets common to all three variants of mouse Fyn (NCBI Gene ID: 14360) were selected from which sense and antisense oligonucleotides were synthesized (Invitrogen). RNAs were transcribed from these oligonucleotides, annealed to form double-stranded siRNA, and purified according to the manufacturer's instructions (Ambion, Austin, TX). Two pairs of non-specific, non-silencing scramble (Scr) oligonucleotides were also designed. Each pair of siFyn (or Scr) oligonucleotides were then extended at their 5′ end with short complementary (dA)6/(dT)6 overhangs (Table S1) to generate sticky-siRNA (ssiRNA). ssiRNAs are a new class of siRNAs designed to improve stability, delivery and effectiveness [59]. MLMVEC were grown to 70% confluency in 12-well or ECIS culture plates and then serum-starved for 1 hour in low serum media (DMEM Low Glucose (Gibco) containing 1% FBS Heat Inactivated (Sigma)). For the 12-well preparations, this was followed by incubation with 100 pmoles (50 pmoles of each ssiRNA) of ssiFyn or ssScr. For ECIS experiments, 60 pmoles (30 pmoles of each construct) ssiRNAs were transfected per well. All transfections utilized Lipofectamine 2000 (Invitrogen) as a carrier. After 6 hours, complete media was added (DMEM supplemented with 20% FBS) for 48 hours before beginning each experiment.

### Western blot analysis

Immunoblots for catalase (1∶6000, Calbiochem), glutathione peroxidase 1 (Gpx-1) (1 µg/ml, Abcam), manganese superoxide dismutase (MnSOD) (1∶100, Santa Cruz), Fyn (1:2000, Santa Cruz), P^Y417^-Fyn (1∶1000, BD) and P^Y528^-Fyn (1∶1000, BD) in MLMVEC or whole lung were performed as previously described [60], [61]. Briefly, MLMVECs were homogenized in lysis buffer containing 150 mM NaCl, 1% Triton X-100, 20 mM Tris-HCl (pH 7.4), 100× protease inhibitor cocktail, 1 mM sodium vanadate, 1 mM NaF, 1 mM PMSF (all from Sigma) for 30 minutes on ice. Lungs were homogenized in 500 μl of tissue protein extraction reagent (Thermo Scientific) with added 100× protease inhibitor cocktail, 1 mM Na_3_VO_4_, 1 mM NaF, and 1 mM PMSF in a FastPrep tubes containing lysing matrix beads (MP Biomedicals). The homogenates were centrifuged, and the supernatants were assayed for protein concentration by using a BCA protein assay kit (Pierce). Soluble proteins (for lung homogenates 40 μg; for cell homogenates 30 or 60 μg) were resolved on an 8% SDS-PAGE (Bio-Rad) and transferred to polyvinylidene fluoride membrane using the iBlot transfer system (Invitrogen). The membranes were blocked with 5% BSA in Tris-buffered saline with 0.1% Tween-20 for 1 hour and then incubated with primary and secondary horseradish peroxidase (HRP)-conjugated antibodies as previously described. Equal protein loading was confirmed by stripping the membranes (Restore Western Blot Stripping buffer, Thermo Scientific) and reprobing with HRP-conjugated anti-Actin (1∶1,000, Abcam) or anti-GAPDH (1∶25,000) antibodies. The blots were developed with regular or enhanced chemiluminescence (ECL or ECLplus; Amersham) and exposed to X-ray film (Kodak BioMax MR). Bands of interest were quantified by densitometric quantification using UN-SCAN-IT gel automated digitizing system software version 5.1 (Silk Scientific).

### Statistics

The effect of genotype on iRBC sequestration in malaria-infected lungs and the comparison of ROS generation by RBC and iRBC were analyzed by unpaired t tests. The effects of malaria and genotype on pulmonary vascular permeability, H_2_O_2_ scavenging and antioxidant or Fyn protein expression were analyzed by a 2-factor (genotype, malaria infection) ANOVA. TER data were analyzed by either 2-factor (treatment, time) or 3-factor (genotype, treatment and time) ANOVA with one repeated measure. Non-normal data were converted to logarithms before analysis. If logarithmic conversion proved insufficient to generate a normal distribution by Kolmogorov–Smirnov testing, nonparametric analysis was used. When a significant interaction F ratio was obtained, least significant differences were calculated to allow comparison of individual means. Values presented in the text are means ± SE. Differences were considered significant when P≤0.05.

## Acknowledgments

The authors would like to thank Maria Febrraio for providing the CD36−/− animals, MR4 for providing the *P. berghei* ANKA parasites, Dr. Volker Heussler for providing tdTomato-transgenic *P. berghei* ANKA and Matt Craig for reviewing the manuscript.

## Supporting Information

Figure S1 **Time course of body weight, temperature and parasitemia kinetics are similar in WT, CD36−/− and Fyn−/− mice.** The change in body weight (A), body temperature (B) and parasitemia (C) of mice after receiving an intraperitoneal injection of 10^6^ *P. berghei* ANKA iRBCs. Weight and temperature are expressed as the percentage change from the weight or temperature at day 0. Data are presented as the means ± SE (n = 10–24).
(TIF)

Figure S2 **Flow cytometric assessment of parasite lung accumulation in WT and CD36−/− mice.** **A**) C57BL/6J WT and CD36−/− mice were infected with a transgenic strain of *P. berghei* ANKA that expressed the red fluorescent protein tdTomato (PbA tdT). Lungs were perfused and isolated on day 4 post-infection and sorted on tdTomato+ cells. The data are representative of the results from 9 animals per group. Data were acquired by running samples on a FACS Calibur flow cytometer using CellQuest software (BD Biosciences, Mountain View, CA, USA). Data were analyzed using FloJo software (Tree Star, Inc., Ashland, OR, USA). **B**) The number of PbAtdT-positive lung cells in WT and CD36−/− lungs day 4 post-infection as determined by flow cytometry. Data are presented as the means ± SE of three independent experiments (n = 9 mice per group) (*P*<0.001).
(TIF)

Table S1 **Fyn ssiRNA oligonucleotide template sequences.** Fyn-specific target (FYN) and non-specific, non-silencing Scramble (Scr) oligonucleotides sequences of 1A and 2A are shown. Each pair of small-interfering Fyn (siFYN) or small Scramble (sScr) oligonucleotides were extended at their 5′ end with short complementary (dA)6/(dT)6 overhangs to generate sticky-siFYN (ssiFYN) or sticky-sScr (ssScr) for concatemerization. Further, all oligonucleotides were extended at their 3′ end with 8 nt leader sequences that are complementary to T7 promoter primer for RNA synthesis.
(DOCX)

## References

[1] Mohan A, Sharma SK, Bollineni S (2008) Acute lung injury and acute respiratory distress syndrome in malaria. J Vector Borne Dis 45: 179–193. 18807374

[2] Taylor WR, Canon V, White NJ (2006) Pulmonary manifestations of malaria: recognition and management. Treat Respir Med 5: 419–428. 17154671 10.2165/00151829-200605060-00007

[3] Gruring C, Heiber A, Kruse F, Ungefehr J, Gilberger TW, et al (2011) Development and host cell modifications of Plasmodium falciparum blood stages in four dimensions. Nat Commun 2: 165 ncomms1169 [pii];10.1038/ncomms1169 [doi]. 21266965 10.1038/ncomms1169

[4] Miller LH, Baruch DI, Marsh K, Doumbo OK (2002) The pathogenic basis of malaria. Nature 415: 673–679. 11832955 10.1038/415673a

[5] Sherman IW, Eda S, Winograd E (2003) Cytoadherence and sequestration in Plasmodium falciparum: defining the ties that bind. Microbes and Infection 5: 897–909. 12919858 10.1016/s1286-4579(03)00162-x

[6] Gamain B, Smith JD, Viebig NK, Gysin J, Scherf A (2007) Pregnancy-associated malaria: parasite binding, natural immunity and vaccine development. Int J Parasitol 37: 273–283 S0020–7519(06)00403–6 [pii]; 10.1016/j.ijpara.2006.11.011 [doi] 17224156 10.1016/j.ijpara.2006.11.011

[7] Haldar K, Murphy SC, Milner DA, Taylor TE (2007) Malaria: mechanisms of erythrocytic infection and pathological correlates of severe disease. Annu Rev Pathol 2: 217–249 10.1146/annurev.pathol.2.010506.091913 [doi] 18039099 10.1146/annurev.pathol.2.010506.091913

[8] Seydel KB, Milner DA Jr, Kamiza SB, Molyneux ME, Taylor TE (2006) The distribution and intensity of parasite sequestration in comatose Malawian children. J Infect Dis 194: 208–5 JID35876 [pii]; 10.1086/505078 [doi] 16779727 10.1086/505078 PMC1515074

[9] Fonager J, Pasini EM, Braks JA, Klop O, Ramesar J, et al (2012) Reduced CD36-dependent tissue sequestration of Plasmodium-infected erythrocytes is detrimental to malaria parasite growth in vivo. J Exp Med 209: 93–107. 22184632 10.1084/jem.20110762 PMC3260870

[10] Franke-Fayard B, Janse CJ, Cunha-Rodrigues M, Ramesar J, Buscher P, et al (2005) Murine malaria parasite sequestration: CD36 is the major receptor, but cerebral pathology is unlinked to sequestration. Proc Natl Acad Sci U S A 102: 11468–11473. 16051702 10.1073/pnas.0503386102 PMC1183563

[11] Lovegrove FE, Gharib SA, Pena-Castillo L, Patel SN, Ruzinski JT, et al (2008) Parasite burden and CD36-mediated sequestration are determinants of acute lung injury in an experimental malaria model. PLoS Pathog 4: e1000068. 18483551 10.1371/journal.ppat.1000068 PMC2364663

[12] van der Heyde HC, Bauer P, Sun G, Chang WL, Yin L, et al (2001) Assessing vascular permeability during experimental cerebral malaria by a radiolabeled monoclonal antibody technique. Infect Immun 69: 3460–3465. 11292776 10.1128/IAI.69.5.3460-3465.2001 PMC98312

[13] Chang WL, Jones SP, Lefer DJ, Welbourne T, Sun G, et al (2001) CD8(+)-T-cell depletion ameliorates circulatory shock in Plasmodium berghei-infected mice. Infect Immun 69: 7341–7348. 11705906 10.1128/IAI.69.12.7341-7348.2001 PMC98820

[14] Janse CJ, Waters AP (1995) Plasmodium berghei: the application of cultivation and purification techniques to molecular studies of malaria parasites. Parasitol Today 11: 138–143. 0169–4758(95)80133–2 [pii]. 10.1016/0169-4758(95)80133-2 15275357

[15] Mons B, Janse CJ, Boorsma EG, Van der Kaay HJ (1985) Synchronized erythrocytic schizogony and gametocytogenesis of Plasmodium berghei in vivo and in vitro. Parasitology 91 (Pt 3): 423–430. 10.1017/s0031182000062673 3909068

[16] Cunha-Rodrigues M, Portugal S, Febbraio M, Mota MM (2007) Bone marrow chimeric mice reveal a dual role for CD36 in Plasmodium berghei ANKA infection. Malar J 6: 32 1475–2875-6–32 [pii]; –––10.1186/1475–2875–6–32 [doi] 17367535 10.1186/1475-2875-6-32 PMC1832198

[17] Bull HA, Brickell PM, Dowd PM (1994) Src-related protein tyrosine kinases are physically associated with the surface antigen CD36 in human dermal microvascular endothelial cells. FEBS Lett 351: 41–44. 7521304 10.1016/0014-5793(94)00814-0

[18] Jimenez B, Volpert OV, Crawford SE, Febbraio M, Silverstein RL, et al (2000) Signals leading to apoptosis-dependent inhibition of neovascularization by thrombospondin-1. Nat Med 6: 41–48. 10613822 10.1038/71517

[19] Michel CC, Curry FE (1999) Microvascular permeability. Physiol Rev 79: 703–761. 10390517 10.1152/physrev.1999.79.3.703

[20] Pearse DB, Becker PM, Permutt S (2001) Effect of changing vascular volume on measurement of protein reflection coefficient in ischemic lungs. Am J Physiol Heart Circ Physiol 280: H918–H924. 11158994 10.1152/ajpheart.2001.280.2.H918

[21] Davis SP, Amrein M, Gillrie MR, Lee K, Muruve DA, et al (2012) Plasmodium falciparum-induced CD36 clustering rapidly strengthens cytoadherence via p130CAS-mediated actin cytoskeletal rearrangement. FASEB J 26: 1119–1130 fj.11–196923 [pii]; –10.1096/fj.11–196923 [doi] 22106368 10.1096/fj.11-196923 PMC3289509

[22] Han J, Shuvaev VV, Muzykantov VR (2011) Catalase and superoxide dismutase conjugated with platelet-endothelial cell adhesion molecule antibody distinctly alleviate abnormal endothelial permeability caused by exogenous reactive oxygen species and vascular endothelial growth factor. J Pharmacol Exp Ther 338: 82–91 jpet.111.180620 [pii]; 10.1124/jpet.111.180620 [doi] 21474567 10.1124/jpet.111.180620 PMC3126647

[23] Pearse DB, Shimoda LA, Verin AD, Bogatcheva N, Moon C, et al (2003) Effect of cGMP on lung microvascular endothelial barrier dysfunction following hydrogen peroxide. Endothelium 10: 309–317. 14741846 10.1080/10623320390272307

[24] Atamna H, Ginsburg H (1993) Origin of reactive oxygen species in erythrocytes infected with Plasmodium falciparum. Mol Biochem Parasitol 61: 231–241. 8264727 10.1016/0166-6851(93)90069-a

[25] Butzloff S, Groves MR, Wrenger C, Muller IB (2012) Cytometric quantification of singlet oxygen in the human malaria parasite Plasmodium falciparum. Cytometry A 81: 698–703 10.1002/cyto.a.22081 [doi] 22736452 10.1002/cyto.a.22081

[26] Mishra NC, Kabilan L, Sharma A (1994) Oxidative stress and malaria-infected erythrocytes. Indian J Malariol 31: 77–87. 7713262

[27] Pino P, Vouldoukis I, Kolb JP, Mahmoudi N, Sportes-Livage I, et al (2003) Plasmodium falciparum--infected erythrocyte adhesion induces caspase activation and apoptosis in human endothelial cells. J Infect Dis 187: 1283–1290. 12696008 10.1086/373992

[28] Postma NS, Mommers EC, Eling WM, Zuidema J (1996) Oxidative stress in malaria; implications for prevention and therapy. Pharm World Sci 18: 121–129. 8873227 10.1007/BF00717727

[29] Cambos M, Bazinet S, Abed E, Sanchez-Dardon J, Bernard C, et al (2010) The IL-12p70/IL-10 interplay is differentially regulated by free heme and hemozoin in murine bone-marrow-derived macrophages. Int J Parasitol 40: 1003–1012 S0020–7519(10)00065–2 [pii]; 10.1016/j.ijpara.2010.02.007 [doi] 20211185 10.1016/j.ijpara.2010.02.007

[30] Qin Y, Lu M, Gong X (2008) Dihydrorhodamine 123 is superior to 2,7-dichlorodihydrofluorescein diacetate and dihydrorhodamine 6G in detecting intracellular hydrogen peroxide in tumor cells. Cell Biology International 32: 224–228 doi: 10.1016/j.cellbi.2007.08.028 17920943 10.1016/j.cellbi.2007.08.028

[31] Sakurada H, Koizumi H, Ohkawara A, Ueda T, Kamo N (1992) Use of dihydrorhodamine 123 for detecting intracellular generation of peroxides upon UV irradiation in epidermal keratinocytes. Arch Dermatol Res 284: 114–116. 1610212 10.1007/BF00373382

[32] Cox-Singh J, Hiu J, Lucas SB, Divis PC, Zulkarnaen M, et al (2010) Severe malaria – a case of fatal Plasmodium knowlesi infection with post-mortem findings: a case report. Malar J 9: 10 1475–2875–9–10 [pii]; –––10.1186/1475–2875–9–10 [doi] 20064229 10.1186/1475-2875-9-10 PMC2818646

[33] Mota MM, Jarra W, Hirst E, Patnaik PK, Holder AA (2000) Plasmodium chabaudi-infected erythrocytes adhere to CD36 and bind to microvascular endothelial cells in an organ-specific way. Infect Immun 68: 4135–4144. 10858230 10.1128/iai.68.7.4135-4144.2000 PMC101711

[34] Smith JD, Kyes S, Craig AG, Fagan T, Hudson-Taylor D, et al (1998) Analysis of adhesive domains from the A4VAR Plasmodium falciparum erythrocyte membrane protein-1 identifies a CD36 binding domain. Mol Biochem Parasitol 97: 133–148. 9879893 10.1016/s0166-6851(98)00145-5

[35] Febbraio M, Hajjar DP, Silverstein RL (2001) CD36: a class B scavenger receptor involved in angiogenesis, atherosclerosis, inflammation, and lipid metabolism. J Clin Invest 108: 785–791. 11560944 10.1172/JCI14006 PMC200943

[36] Curry FR, Adamson RH (2010) Vascular permeability modulation at the cell, microvessel, or whole organ level: towards closing gaps in our knowledge. Cardiovasc Res 87: 218–229. 20418473 10.1093/cvr/cvq115 PMC2895542

[37] Schmidt EP, Yang Y, Janssen WJ, Gandjeva A, Perez MJ, et al.. (2012) The pulmonary endothelial glycocalyx regulates neutrophil adhesion and lung injury during experimental sepsis. Nat Med. nm.2843 [pii]; 10.1038/nm.2843 [doi]. 10.1038/nm.2843 PMC3723751 22820644

[38] Moldobaeva A, Welsh-Servinsky LE, Shimoda LA, Stephens RS, Verin AD, et al (2006) Role of protein kinase G in barrier-protective effects of cGMP in human pulmonary artery endothelial cells. Am J Physiol Lung Cell Mol Physiol 290: L919–L930. 16339778 10.1152/ajplung.00434.2005

[39] Sponaas AM, Freitas do Rosario AP, Voisine C, Mastelic B, Thompson J, et al (2009) Migrating monocytes recruited to the spleen play an important role in control of blood stage malaria. Blood 114: 5522–5531. 19837977 10.1182/blood-2009-04-217489

[40] Deroost K, Tyberghein A, Lays N, Noppen S, Schwarzer E, et al (2013) Hemozoin induces lung inflammation and correlates with malaria-associated acute respiratory distress syndrome. Am J Respir Cell Mol Biol 48: 589–600 rcmb.2012–0450OC [pii]; –10.1165/rcmb.2012–0450OC [doi] 23328641 10.1165/rcmb.2012-0450OC

[41] Stevenson MM, Riley EM (2004) Innate immunity to malaria. Nat Rev Immunol 4: 169–180 10.1038/nri1311 [doi];nri1311 [pii] 15039754 10.1038/nri1311

[42] Gillrie MR, Krishnegowda G, Lee K, Buret AG, Robbins SM, et al (2007) Src-family kinase dependent disruption of endothelial barrier function by Plasmodium falciparum merozoite proteins. Blood 110: 3426–3435. 17693580 10.1182/blood-2007-04-084582 PMC2200906

[43] Gillrie MR, Lee K, Gowda DC, Davis SP, Monestier M, et al (2012) Plasmodium falciparum histones induce endothelial proinflammatory response and barrier dysfunction. Am J Pathol 180: 1028–1039. 22260922 10.1016/j.ajpath.2011.11.037 PMC3448071

[44] Hu G, Minshall RD (2009) Regulation of transendothelial permeability by Src kinase. Microvasc Res 77: 21–25. 19027754 10.1016/j.mvr.2008.10.002

[45] Frame MC, Fincham VJ, Carragher NO, Wyke JA (2002) v-Src's hold over actin and cell adhesions. Nat Rev Mol Cell Biol 3: 233–245. 11994743 10.1038/nrm779

[46] Giannoni E, Buricchi F, Raugei G, Ramponi G, Chiarugi P (2005) Intracellular reactive oxygen species activate Src tyrosine kinase during cell adhesion and anchorage-dependent cell growth. Mol Cell Biol 25: 6391–6403. 16024778 10.1128/MCB.25.15.6391-6403.2005 PMC1190365

[47] Appleby MW, Gross JA, Cooke MP, Levin SD, Qian X, et al.. (1992) Defective T cell receptor signaling in mice lacking the thymic isoform of p59fyn. Cell 70: 751–763. 0092–8674(92)90309–Z [pii]. 10.1016/0092-8674(92)90309-z 1516132

[48] Filby A, Seddon B, Kleczkowska J, Salmond R, Tomlinson P, et al.. (2007) Fyn regulates the duration of TCR engagement needed for commitment to effector function. J Immunol 179: 4635–4644. 179/7/4635 [pii]. 10.4049/jimmunol.179.7.4635 17878361

[49] Gadue P, Morton N, Stein PL (1999) The Src family tyrosine kinase Fyn regulates natural killer T cell development. J Exp Med 190: 1189–1196. 10523617 10.1084/jem.190.8.1189 PMC2195667

[50] Stein PL, Lee HM, Rich S, Soriano P (1992) pp59fyn mutant mice display differential signaling in thymocytes and peripheral T cells. Cell 70: 741–750. 0092–8674(92)90308–Y [pii]. 10.1016/0092-8674(92)90308-y 1387588

[51] Febbraio M, Abumrad NA, Hajjar DP, Sharma K, Cheng W, et al (1999) A null mutation in murine CD36 reveals an important role in fatty acid and lipoprotein metabolism. J Biol Chem 274: 19055–19062. 10383407 10.1074/jbc.274.27.19055

[52] Graewe S, Retzlaff S, Struck N, Janse CJ, Heussler VT (2009) Going live: a comparative analysis of the suitability of the RFP derivatives RedStar, mCherry and tdTomato for intravital and in vitro live imaging of Plasmodium parasites. Biotechnol J 4: 895–902. 19492329 10.1002/biot.200900035

[53] Rentsendorj O, Damarla M, Aggarwal NR, Choi JY, Johnston L, et al (2011) Knockdown of lung phosphodiesterase 2A attenuates alveolar inflammation and protein leak in a two-hit mouse model of acute lung injury. Am J Physiol Lung Cell Mol Physiol 301: L161–L170. 21571906 10.1152/ajplung.00073.2011 PMC3154628

[54] Siracusa MC, Reece JJ, Urban JF Jr, Scott AL (2008) Dynamics of lung macrophage activation in response to helminth infection. J Leukoc Biol 84: 1422–1433. 18719016 10.1189/jlb.0308199 PMC2614596

[55] Dodd-o JM, Hristopoulos ML, Kibler K, Gutkowska J, Mukaddam-Daher S, et al (2008) The role of natriuretic peptide receptor-A signaling in unilateral lung ischemia-reperfusion injury in the intact mouse. Am J Physiol Lung Cell Mol Physiol 294: L714–L723. 18223163 10.1152/ajplung.00185.2007

[56] Pearse DB, Sylvester JT (1996) Vascular injury in isolated sheep lungs. Role of ischemia, extracorporeal perfusion, and oxygen. Am J Respir Crit Care Med 153: 196–202. 8542116 10.1164/ajrccm.153.1.8542116

[57] Moldobaeva A, Wagner EM (2005) Difference in proangiogenic potential of systemic and pulmonary endothelium: role of CXCR2. Am J Physiol Lung Cell Mol Physiol 288: L1117–L1123. 15722378 10.1152/ajplung.00370.2004

[58] Stephens RS, Rentsendorj O, Servinsky LE, Moldobaeva A, Damico R, et al (2010) cGMP increases antioxidant function and attenuates oxidant cell death in mouse lung microvascular endothelial cells by a protein kinase G-dependent mechanism. Am J Physiol Lung Cell Mol Physiol 299: L323–L333. 20453163 10.1152/ajplung.00442.2009 PMC2951066

[59] Bolcato-Bellemin AL, Bonnet ME, Creusat G, Erbacher P, Behr JP (2007) Sticky overhangs enhance siRNA-mediated gene silencing. Proc Natl Acad Sci U S A 104: 16050–16055. 17913877 10.1073/pnas.0707831104 PMC2042160

[60] Rentsendorj O, Mirzapoiazova T, Adyshev D, Servinsky LE, Renne T, et al (2008) Role of vasodilator-stimulated phosphoprotein in cGMP-mediated protection of human pulmonary artery endothelial barrier function. Am J Physiol Lung Cell Mol Physiol 294: L686–L697. 18281604 10.1152/ajplung.00417.2007

[61] Schmidt EP, Damarla M, Rentsendorj O, Servinsky LE, Zhu B, et al (2008) Soluble guanylyl cyclase contributes to ventilator-induced lung injury in mice. Am J Physiol Lung Cell Mol Physiol 295: L1056–L1065. 18849438 10.1152/ajplung.90329.2008 PMC2604795

