# Supplementary figures and images for: CD36 and Fyn Kinase Mediate Malaria-Induced Lung Endothelial Barrier Dysfunction in Mice Infected with Plasmodium berghei
Source: PLoS One. 2013 Aug 15;8(8):e71010. doi: 10.1371/journal.pone.0071010 (PMC3744507; doi:10.1371/journal.pone.0071010)

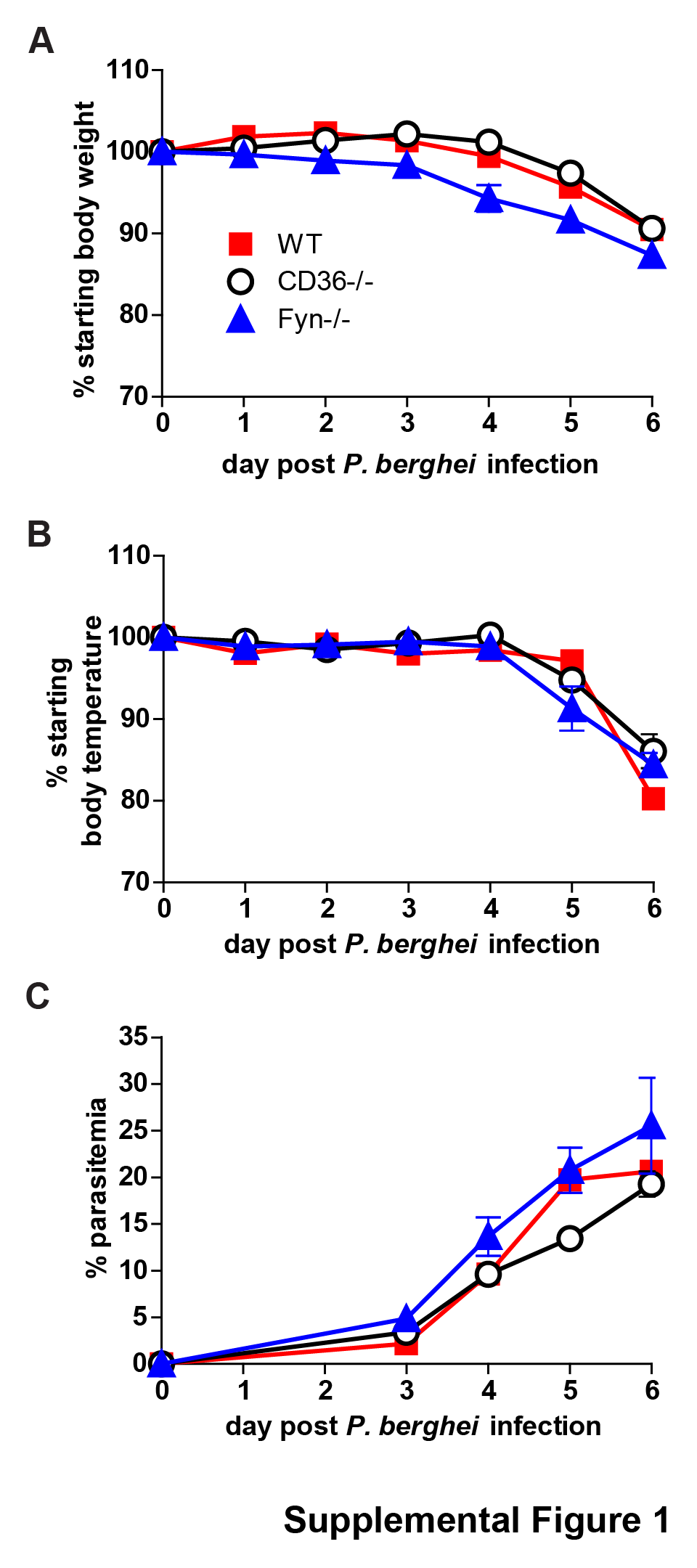

Supplement: Figure S1 — Time course of body weight, temperature and parasitemia kinetics are similar in WT, CD36−/− and Fyn−/− mice. The change in body weight (A), body temperature (B) and parasitemia (C) of mice after receiving an intraperitoneal injection of 106 P. berghei ANKA iRBCs. Weight and temperature are expressed as the percentage change from the weight or temperature at day 0. Data are presented as the means ± SE (n = 10–24). (TIF) [file pone.0071010.s001.tif]

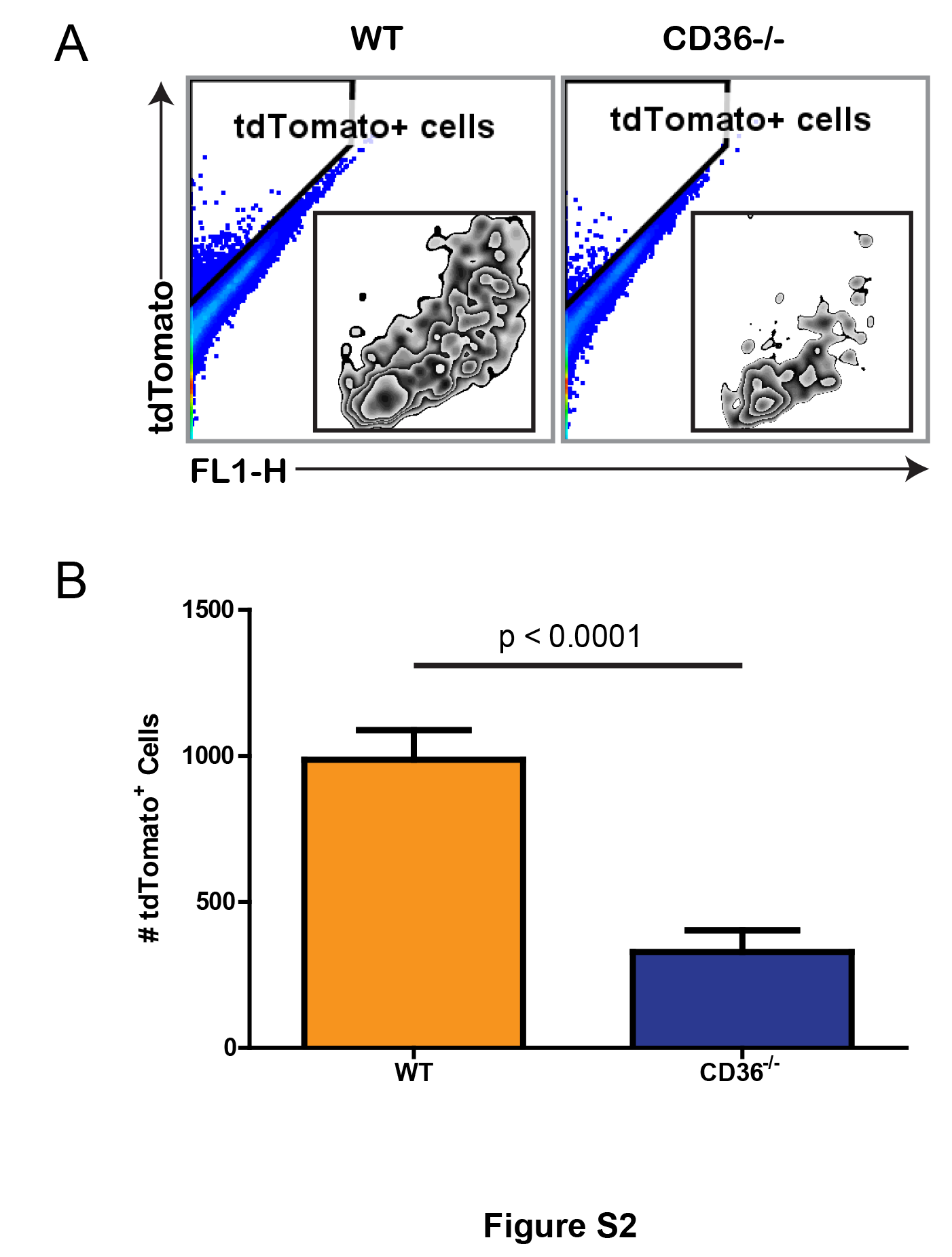

Supplement: Figure S2 — Flow cytometric assessment of parasite lung accumulation in WT and CD36−/− mice. A) C57BL/6J WT and CD36−/− mice were infected with a transgenic strain of P. berghei ANKA that expressed the red fluorescent protein tdTomato (PbA tdT). Lungs were perfused and isolated on day 4 post-infection and sorted on tdTomato+ cells. The data are representative of the results from 9 animals per group. Data were acquired by running samples on a FACS Calibur flow cytometer using CellQuest software (BD Biosciences, Mountain View, CA, USA). Data were analyzed using FloJo software (Tree Star, Inc., Ashland, OR, USA). B) The number of PbAtdT-positive lung cells in WT and CD36−/− lungs day 4 post-infection as determined by flow cytometry. Data are presented as the means ± SE of three independent experiments (n = 9 mice per group) (P<0.001). (TIF) [file pone.0071010.s002.tif]
