# Supplementary material for: CD36 and Fyn Kinase Mediate Malaria-Induced Lung Endothelial Barrier Dysfunction in Mice Infected with Plasmodium berghei
Source: PLoS One. 2013 Aug 15;8(8):e71010. doi: 10.1371/journal.pone.0071010 (PMC3744507; doi:10.1371/journal.pone.0071010)
Supplement: Table S1 — Fyn ssiRNA oligonucleotide template sequences. Fyn-specific target (FYN) and non-specific, non-silencing Scramble (Scr) oligonucleotides sequences of 1A and 2A are shown. Each pair of small-interfering Fyn (siFYN) or small Scramble (sScr) oligonucleotides were extended at their 5′ end with short complementary (dA)6/(dT)6 overhangs to generate sticky-siFYN (ssiFYN) or sticky-sScr (ssScr) for concatemerization. Further, all oligonucleotides were extended at their 3′ end with 8 nt leader sequences that are complementary to T7 promoter primer for RNA synthesis. (DOCX) [file pone.0071010.s003.docx]

| **Construct Name** | **Sequence** |
| --- | --- |
| ssiFYN 1S | TTTTTTTGCGTGGAAGTTGTTGTAGCCTGTCTC |
| ssiFYN 1A | AAAAAACTACAACAACTTCCACGCACCTGTCTC |
| ssiFYN 2S | TTTTTTGCATCTTTGCGGCCAAGTTCCTGTCTC |
| ssiFYN 2A | AAAAAAAACTTGGCCGCAAAGATGCCCTGTCTC |
| ssiScr 1S | TTTTTTCTTGCGCTTAATTGAGTTCCCTGTCTC |
| ssiScr 1A | AAAAAAGAACTCAATTAAGCGCAAGCCTGTCTC |
| ssiScr 2S | TTTTTTACGTGACACGTTCGGAGAACCTGTCTC |
| ssiScr 2A | AAAAAATTCTCCGAACGTGTCACGTCCTGTCTC |

Table S1: Fyn-specific target (FYN) and non-specific, non-silencing Scramble (Scr) oligonucleotides sequences.
